# Optimization of complex engineering problems using modified sine cosine algorithm

**DOI:** 10.1038/s41598-022-24840-z

**Published:** 2022-11-28

**Authors:** Chao Shang, Ting-ting Zhou, Shuai Liu

**Affiliations:** 1 Pujiang Institute, Nanjing Tech University, Nanjing, 211134 China; 2 School of Civil and Hydraulic Engineering, Huazhong University of Science and Technology, Wuhan, 430074 China

**Keywords:** Civil engineering, Applied mathematics

## Abstract

In this article, a modified version of the Sine Cosine algorithm (MSCA) is proposed to solve the optimization problem. Based on the Sine Cosine algorithm (SCA), the position update formula of SCA is redefined to increase the convergence speed, then the Levy random walk mutation strategy is adopted to improve the population diversity. In order to verify the performance of MSCA, 24 well-known classical benchmark problems and IEEE CEC2017 test suites were introduced, and by comparing MSCA with several popular methods, it is demonstrated that MSCA has good convergence and robustness. Finally, MSCA is used to address six complex engineering design problems, demonstrating the engineering utility of the algorithm.

## Introduction

Optimization is the process of finding the best solution to a problem by specific rules^1^. Traditional optimization techniques need to satisfy the preconditions of continuity and differentiability of the objective function, which makes it impossible to apply anywhere in the real world^2^. The heuristic algorithm treats the optimization problem as a black box, and does not need to consider the problem information, which brings a boon to the many complex optimization problems. In recent years, as an emerging optimization technology, heuristic algorithms have received widespread popularity in the field of engineering^3^. Heuristic algorithms are mainly inspired by natural experience and observations, which obtain beneficial information by simulating the cooperation or competition of individuals in the population to find possible better positions in the search space. In general, heuristic algorithms can be classified into three main types^4^: evolution-based, physics-based and swarm-based algorithms. Evolution-based algorithms are inspired by the laws of biological evolution, individuals exchange information to ensure that they can find a favorable position in the search space. The representative algorithms are Genetic Algorithms (GA)^5^, Differential Evolution (DE)^6^ and Evolutionary Strategies (ES)^7^. Physics-based algorithms inspired by the laws of physics in nature. The most popular methods are Gravitational Search Algorithm (GSA)^8^, Charged System Search (CSS)^9^ and Henry gas solubility optimization(HGSO). Swarm-based algorithm mimic social behaviors such as cooperation, competition, and foraging. The most typical algorithms are Ant Colony Optimization (ACO)^10^, Particle Swarm Optimization (PSO)^11^ and Harris Hawks Optimization (HHO)^12^.

Recently, many algorithms have been proposed and received more attention from many researchers. For example—Sadoun et al. ^13^ have applied the Dwarf Mongoose Optimization algorithm (DMO)^14^ to predict the effect of Al_2_O_3_ nanoparticle content. In^15^, Reptile search Algorithm (RSA)^16^ combines with ant colony optimization was developed for churn prediction. In^17^, Ebola Optimization Search Algorithm (EOSA)^18^ is used to evaluation the performance convolutional neural networks. In^19^, Aquila Optimizer (AO)^20^ is used for weights allocated of forecasting model. In^21^, Discrete Equilibrium Optimizer and Simulated Annealing is hybridized to solved structural optimization and multi-level image segmentation problems. In^22^, Snake Optimizer (SO) is proposed to tackle real-world engineering problems. In^23^, several modified versions of HHO were comprehensively reviewed for engineering application. In^24^, an Enhanced Remora Optimization Algorithm (EROA) is developed for constrained engineering problems. In^25^, a modified COOT algorithm is presented to the dimensionality reduction problem. In^26^, a modified version of Aquila Optimizer (MAO) is developed to solve CEC2017 test suit and five different engineering problems. In^27^, an Improved Wild Horse Optimizer (IWHO) is proposed to solve high-dimensional cases. In^28^, a self‑adaptive Harris Hawks optimization algorithm with opposition‑based learning and chaotic local search strategy is used to solve constrained problems. In^29^, Arithmetic Optimization Algorithm (AOA) is used for the image fusion process.

Sine Cosine Algorithm (SCA)is a swarm-based optimization algorithm that evolves with the help of mathematical models^30^. The SCA algorithm via a set of random solutions as the beginning, which perfectly avoids the defect that other classical algorithms fall into local optimum in the early search process of optimization. During the search process, periodic sine and cosine trigonometric functions were adopted to adaptively modify the search range to ensure that individuals can fluctuate toward or away from the global optimum^31^. At the same time, the currently found global best position in the population is continuously updated to improve the ability of individual to move to the optimal position. In^2^, SCA was tested on several different types of benchmark problems and successfully optimized the cross-section of an aircraft wing. In^32^, SCA is mixed with Salp Swarm Algorithm (SSA) for feature selection problems. In^33^, an advanced sine cosine algorithm is employed for assign the weight of ensemble prediction model. In^34^, a modified version of the SCA is proposed for training multilayer perceptron. In^35^, a dynamic sine cosine algorithm is employed for solve large scale global optimization problems. In^36^, a hybrid self-adaptive sine cosine algorithm is proposed to solve engineering application problems.

Although many experts employ different strategies to strive to improve its performance of original SCA, it can be seen from the optimization metrics obtained by the improved version on the test problem that in some cases, SCA exhibits low convergence and inability to jump out of local optima.

Therefore, the aim of this paper is to propose a modified version of the SCA named MSCA. The original formula of SCA is adjusted, which effectively avoids the defect of slow convergence, and improves the search range and optimization ability of the algorithm. After that, while retaining the excellent individuals, the personal best position and the Levy flight strategy in the cuckoo are introduced into the mutation operator to ensure the individual diversity.

To prove the superior performance of MSCA on optimization problems, the proposed method has undergone preliminary performance tests on the classic benchmark functions and the CEC2017 test set. Furthermore, to demonstrate the engineering practicality, the proposed method is experimented on complex engineering problems. Compared with the existing advanced algorithms, the proposed method can obtain more reliable results.

The rest of the article is organized as follows: Section “The modified sine cosine algorithm” briefly introduces the Sine Cosine Algorithm (SCA) and proposes a modified version of Sine Cosine Algorithm (MSCA). In Section “MSCA for numerical optimization problems”, 24 classical benchmark functions and CEC2017 test suites are adopted for numerical experiments to prove search performance of MSCA. Six classical engineering optimization problems are adopted to verify the engineering practicability of MSCA in Section “MSCA for engineering optimization problems”. Finally, Section “Conclusion” summarizes the work of this article.

## The modified sine cosine algorithm

### Brief introduction of SCA

**Figure 1:**
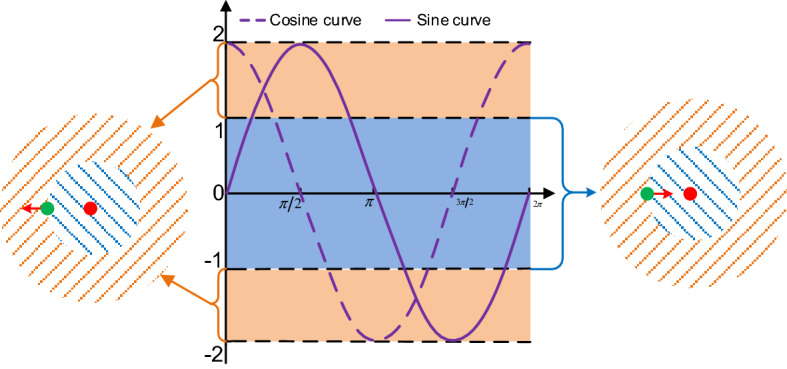
Diagram of SCA search mode.

SCA is a swarm-based optimization method proposed by Mirjalili in 2016^2^. In the optimization process of SCA, all solutions find the best possible position in the search space according to the pattern of sine and cosine random cycles. Same as other algorithm optimization stages, the exploration phase with high disturbance and the development phase with low randomness constitute the optimization process of SCA. The diagram of SCA search mode as shown in Fig. 1, and the two-phases position update equation of SCA is shown as follows:

1 $$X_{ij\;}^{k + 1} = \left\{ {_{{X_{ij\;}^{\;k} + r_{1} \times \cos \left( {r_{2} } \right) \times \left| {r_{3} \times P_{j}^{k} - X_{ij\;}^{\;k} } \right|,\;\begin{array}{*{20}c} {r_{4} \ge 0.5} \\ \end{array} }}^{{X_{ij\;}^{\;k} + r_{1} \times \sin \;\left( {r_{2} } \right) \times \left| {r_{3} \times P_{j}^{k} - X_{ij\;}^{\;k} } \right|,\;\begin{array}{*{20}c} {r_{4} < 0.5} \\ \end{array} }} } \right.$$

where $X_{ij\;}^{k + 1}$ denotes the position of *i*th individual in *j*th dimension at (*k* + 1)th iteration. $P_{j}^{k}$ denotes the global best position in *j*th dimension at *k*th iteration. The parameter $r_{1}$ decreases linearly with the iterative process, which is used to ensure the balance between exploration and exploitation, the $r_{1}$ is defined as follows:

2 $$r_{1} = a - k\frac{a}{{\overline{k} }}$$

where $a$ is a constant number, and $\overline{k}$ is the maximum number of iterations. $r_{2}$ is the random number uniformly distributed in [0, 2*π*], $r_{3}$ is the random number uniformly distributed in [0,2], when $r_{3} > 1$, The exchange of information between $P_{j}^{k}$ and $X_{ij\;}^{\;k}$ increases; when $r_{3} < 1$, the influence between $P_{j}^{k}$ and $X_{ij\;}^{\;k}$ is reduced. $r_{4}$ is a random number in the interval [0,1], which is used to switch with equal probability between sine cosine trigonometric functions.

### The MSCA algorithm

In this section, a modified version of SCA is proposed to affronts the drawbacks of SCA exhibits optimization stagnation and slow convergence.

#### Modified position updating of SCA

In the original SCA, only the global optimal position is multiplied by a random factor, which only ensures that the population performs a local search to the current individual position, and ignores the existence of better potential solutions near the global optimal position.

In order to improve the convergence rate and ensure the search balance between the global optimal position and the current individual position, the update formula of SCA is reconstructed. When $r_{4}$ is less than 0.5, a search mode containing a sine function is used to find a potential better solution near the global optimal position. Otherwise, the cosine function formula is adopted to find a better solution near the current individual position. The modified position update as follows:

3 $${\varvec{U}}_{i\;}^{k} = \left\{ {_{{{\varvec{X}}_{i\;}^{\;k} + r_{1} \times \cos \left( {r_{2} } \right) \times \left| {r_{3} \times {\varvec{P}}_{{}}^{k} - {\varvec{X}}_{i\;}^{\;k} } \right|,\begin{array}{*{20}c} {} \\ \end{array} r_{4} \ge 0.5}}^{{{\varvec{X}}_{i\;}^{\;k} + r_{1} \times \sin \;\left( {r_{2} } \right) \times \left| {{\varvec{P}}_{{}}^{k} - r_{3} \times {\varvec{X}}_{i\;}^{\;k} } \right|,\begin{array}{*{20}c} {} \\ \end{array} r_{4} < 0.5}} } \right.$$

where ${\varvec{U}}_{i\;}^{\;k}$ represents the position vector of *i*th temporary individual at *k*th iteration. ${\varvec{X}}_{i\;}^{\;k}$ represents the position vector of *i*th individual at *k*th iteration. $r_{2}$, $r_{3}$ and $r_{4}$ are random numbers for each position vector of *i*th temporary individual. ${\varvec{P}}_{{}}^{k}$ represents the position vector of global best-known position at *k*th iteration.

#### Levy random walk mutation strategy

In SCA, only the global optimal individual is considered to guide the evolution direction of the population. When individual falls into a local optimal, it is difficult to jump out of the local unfavorable position only with the help of the global optimum position, which eventually leads to optimization stagnation.

**Figure 2:**
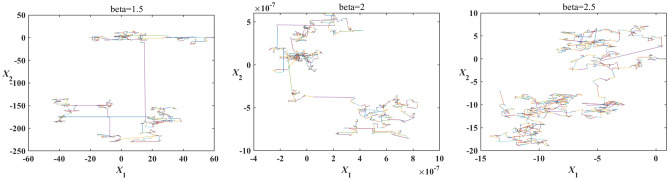
Mantegna simulates Levy flight with different beta values.

Therefore, the personal best position^11^ and the Levy random walk^37^ is introduced into MSCA to make up for the shortcoming of cannot jump out of the local optimal. In order to take full advantage of the mutation operator, the global best position is used to form a difference vector with random individuals to act with the Levy random operator, and finally randomly superimposed on the current position or the personal best position to form a new individual. The Mantegna simulates Levy flight with different beta values as presented in Fig. 2. The expression of ${\varvec{X}}_{i}^{k + 1}$ is defined as follows:

4 $${\varvec{X}}_{i}^{k + 1} = \left\{ {\begin{array}{*{20}l} {{\varvec{U}}_{{r_{5} \;}}^{k} + \left( {{\varvec{P}}^{k} - {\varvec{U}}_{{r_{6} \;}}^{k} } \right) \cdot \user2{\varphi }( - 1,1) \cdot \frac{{\overline{k} - k}}{{\overline{k} }} \cdot {\varvec{Levy}}\left( \beta \right),} \hfill & {r_{7} < 0.5} \hfill \\ {{\varvec{pBest}}_{i\;}^{k} + \left( {{\varvec{P}}^{k} - {\varvec{U}}_{{r_{6} \;}}^{k} } \right) \cdot \user2{\varphi }( - 1,1) \cdot \frac{{\overline{k} - k}}{{\overline{k} }} \cdot {\varvec{Levy}}\left( \beta \right),} \hfill & {r_{7} \ge 0.5} \hfill \\ \end{array} } \right.$$

where ${\varvec{X}}_{i}^{k + 1}$ represents the position vector of *i*th individual at (*k* + 1)th iteration. ${\varvec{pBest}}_{i\;}^{k}$ represents the position vector of personal best position of *i*th individual at *k*th iteration. $r_{5}$ and $r_{6}$ represent the index of randomly selected individuals in the temporary population, $r_{5} \ne r_{6}$. $\user2{\varphi }( - 1,1)$ represents the random vector in the interval [-1,1]. ${\varvec{Levy}}\;\left( \beta \right)$ represents random vector with Levy distribution, $\beta$ is the tuning parameter.

#### Execution steps of MSCA

**Figure 3:**
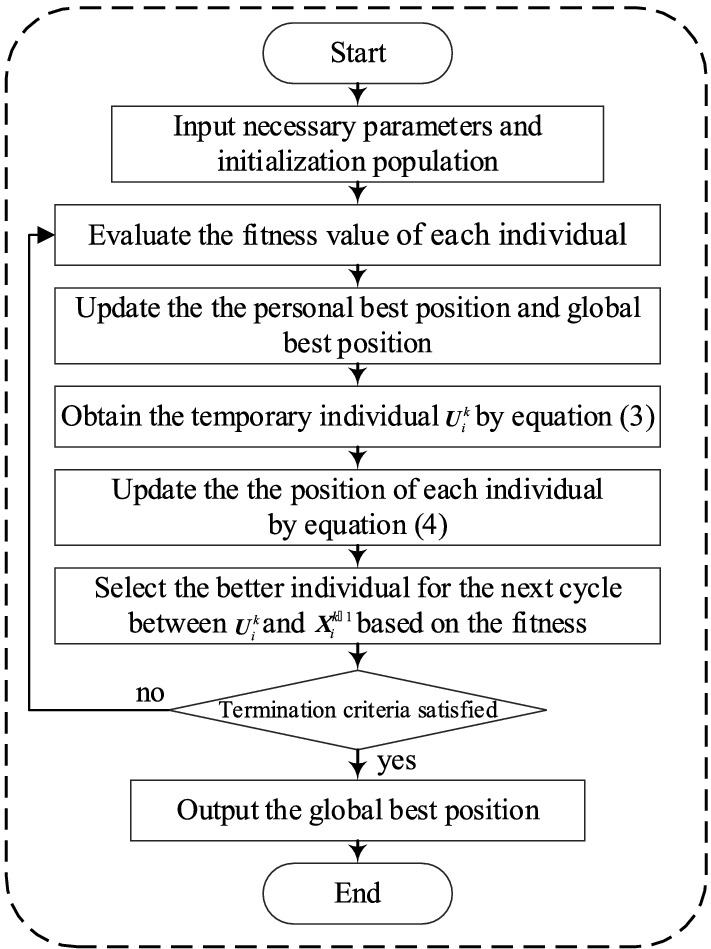
Flowchart of the MSCA method.

The flowchart of the MSCA is shown in Fig. 3:

## MSCA for numerical optimization problems

Compared with the SCA, MSCA has the advantages of better convergence speed and avoiding premature convergence because of the improved position update strategy and the Levy random walk mutation strategy. To further demonstrate the performance of MSCA, 24 classical benchmark problems^38^ and IEEE CEC2017 test suites^39^ are adopted to verify the effectiveness of proposed strategy.

### Benchmark problem set I: classical benchmark problems

**Figure 4:**
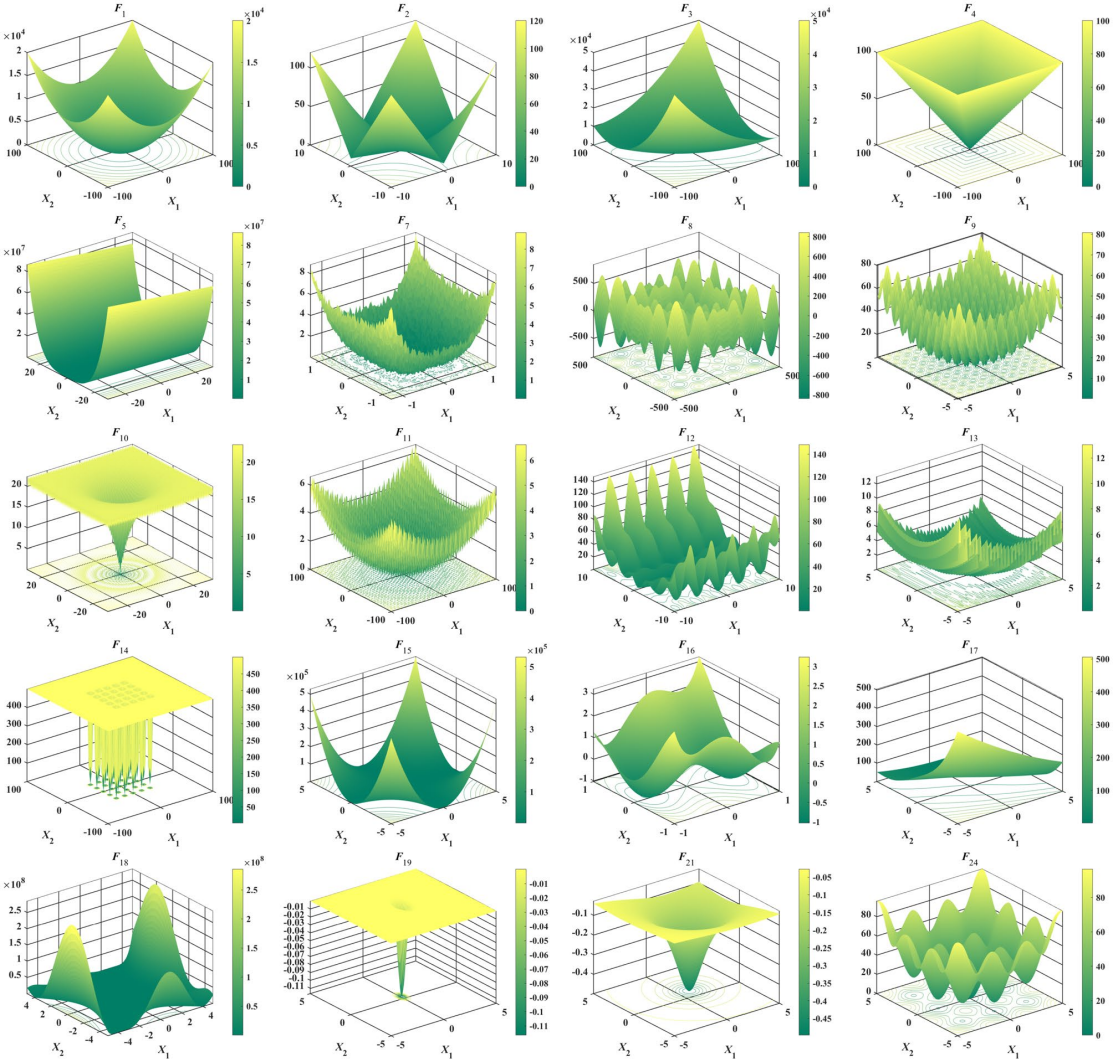
A 2D shape of classical benchmark problems.

The first set of classical benchmark problems can be divided into unimodal (*F*_1_- *F*_6_), multimodal (*F*_7_-*F*_13_) and fixed-dimension multimodal (*F*_14_-*F*_24_). The unimodal was used to validate the convergence performance of the algorithm, the multimodal was used to test the ability of the method to avoid local stagnation, and the fixed-dimension multimodal was designed to examination the balance levels of the algorithm between exploration and exploitation. The 2D shape of the classical problem are listed in Fig. 4. The problems are listed in detail in Table 1. It is worth pointing out that *f*_min_, range and *n* represent the theoretical optimal value, the upper and lower bounds and the dimension of the problem, respectively.

**Table 1:** Details of 24 classical benchmark problems.

Type	Equation	*n*	Range	*f*_min_
Unimodal	$F_{1} (x) = \sum\limits_{i = 1}^{n} {x_{i}^{2} }$	30	[−100, 100]	0
Unimodal	$F_{2} \left( x \right) = \sum\limits_{i = 1}^{n} {\left| {x_{i} } \right|} + \prod\limits_{i = 1}^{n} {\left| {x_{i} } \right|}$	30	[−10, 10]	0
Unimodal	$F_{3} (x) = \sum\limits_{i = 1}^{n} {\left( {\sum\limits_{j = 1}^{i} {x_{j} } } \right)}^{2}$	30	[−100, 100]	0
Unimodal	$F_{4} \left( x \right) = \max \left\{ {\left| {x_{i} } \right|,1 \le i \le n} \right\}$	30	[−100, 100]	0
Unimodal	$F_{5} (x) = \sum\limits_{i = 1}^{n - 1} {[100(x_{i + 1}^{{}} - x_{i}^{2} )^{2} + (x_{i}^{{}} - 1)^{2} ]}$	30	[−30, 30]	0
Unimodal	$F_{6} (x) = \sum\limits_{i = 1}^{n} {(\left| {x_{i} + 0.5} \right|)^{2} }$	30	[−100, 100]	0
Unimodal	$F_{7} (x) = \sum\limits_{i = 1}^{n} {ix_{i}^{4} } + random[0,1)$	30	[−1.28, 1.28]	0
Multimodal	$F_{8} (x) = \sum\limits_{i = 1}^{n} { - x_{i} } \sin (\sqrt {|x_{i} |} )$	30	[−500, 500]	−12,567
Multimodal	$F_{9} (x) = \sum\limits_{i = 1}^{n} {[x_{i}^{2} - 10\cos (2\pi x_{i} ) + 10]}$	30	[−5.12, 5.12]	0
Multimodal	$F_{10} (x) = - 20\exp ( - 0.2\sqrt[{}]{{\frac{1}{n}\sum\limits_{i = 1}^{n} {x_{i}^{2} } }}) - \exp (\frac{1}{n}\sum\limits_{i = 1}^{n} {\cos (2\pi x_{i} ))} + 20 + e$	30	[−32, 32]	0
Multimodal	$F_{11} (x) = \frac{1}{4000}\sum\limits_{i = 1}^{n} {x_{i}^{2} - \prod\limits_{i = 1}^{n} {\cos (\frac{{x_{i} }}{\sqrt i }} } ) + 1$	30	[−600, 600]	0
Multimodal	$$\begin{gathered} F_{12} \left( x \right) = \frac{\pi }{n}\{ 10\sin^{2} (\pi y_{1} ) + \sum\limits_{i = 1}^{n - 1} ( y_{i} - 1)^{2} [1 + 10\sin^{2} (\pi y_{i + 1} )] \hfill \\ \;\;\;\;\;\;\;\;\;\;\; + (y_{n} - 1)^{2} \} \; + \sum\limits_{i = 1}^{n} {u(x_{i} } ,10,100,4) \hfill \\ \;\;\;\;\;\;\;\;y_{i} = 1 + \frac{{x_{i} + 1}}{4},u(x_{i} ,a,k,m) = \left\{ {\begin{array}{*{20}c} {k(x_{i} - a)^{m} \;\;\;\;\;\;x_{i} > a} \\ {0\;\;\;\;\;\;\;\;\;\;\; - a \le x_{i} < a} \\ {\;\;k( - x_{i} - a)^{m} \;\;\;x_{i} < - a} \\ \end{array} } \right. \hfill \\ \end{gathered}$$	30	[−50, 50]	0
Multimodal	$$\begin{gathered} F_{13} (x) = 0.1\{ \sin^{2} (3\pi x_{1} ) + \sum\limits_{i = 1}^{n} {(x_{i} - 1)^{2} [1 + \sin^{2} (3\pi x_{i + 1} + 1)]} \hfill \\ \;\;\;\;\;\;\;\;\;\;\; + (x_{n} - 1)^{2} [1 + \sin^{2} (2\pi x_{n} )]\} + \sum\limits_{i = 1}^{n} {u(x_{i} } ,5,100,4) \hfill \\ \end{gathered}$$	30	[−50, 50]	0
Fixed	$F_{14} (x) = [\frac{1}{500} + \sum\limits_{j = 1}^{25} {\frac{1}{{j + \sum\limits_{i = 1}^{2} {(x_{i} - a_{ij}^{{}} )^{6} } }}} ]^{ - 1}$	2	[−65.536,65.536]	1
Fixed	$F_{15} (x) = \sum\limits_{i = 1}^{11} {\left[ {a_{i} - \frac{{x_{1} (b_{i}^{2} + b_{i}^{{}} x_{2}^{{}} )}}{{b_{i}^{2} + b_{i}^{{}} x_{3}^{{}} + x_{4}^{{}} }}} \right]}^{2}$	4	[−5, 5]	0.0003075
Fixed	$F_{16} (x) = 4x_{1}^{2} - 2.1x_{1}^{4} + \frac{1}{3}x_{1}^{6} + x_{1}^{{}} x_{2}^{{}} - 4x_{2}^{2} + 4x_{2}^{4}$	2	[−5, 5]	−1.0316285
Fixed	$F_{17} (x) = (x_{2}^{{}} - \frac{5.1}{{4\pi^{2} }}x_{1}^{2} + \frac{5}{\pi }x_{1} - 6)^{2} + 10(1 - \frac{1}{8\pi })\cos x_{1} + 10$	2	[−5,10] × [0,15]	0.398
Fixed	$$\begin{gathered} F_{18} (x) = [1 + (x_{1}^{{}} + x_{2}^{{}} + 1)^{2} (19 - 14x_{1}^{{}} + 3x_{1}^{2} - 14x_{2}^{{}} \hfill \\ \;\;\;\;\;\;\;\;\;\;\; + 6x_{1}^{{}} x_{2}^{{}} + 3x_{1}^{2} )] \times [30 + (2x_{1}^{{}} - 3x_{2}^{{}} )^{2} (18 - 32x_{1}^{{}} \hfill \\ \;\;\;\;\;\;\;\;\;\;\; + 12x_{1}^{2} + 48x_{2}^{{}} - 36x_{1}^{{}} x_{2}^{{}} + 27x_{2}^{2} )] \hfill \\ \end{gathered}$$	2	[−2, 2]	3
Fixed	$F_{19} (x) = - \sum\limits_{i = 1}^{4} {c_{i} \exp [ - \sum\limits_{j = 1}^{3} {a_{ij} (x_{j} - p_{ij} )^{2} ]} }$	3	[0,1]	−3.86
Fixed	$F_{20} (x) = - \sum\limits_{i = 1}^{4} {c_{i} \exp [ - \sum\limits_{j = 1}^{6} {a_{ij} (x_{j} - p_{ij} )^{2} ]} }$	6	[0,1]	−3.32
Fixed	$F_{22} (x) = - \sum\limits_{i = 1}^{5} {[(x - a_{i} )(x - a_{i} )^{T} + c_{i} ]^{ - 1} }$	4	[0,10]	−10.1532
Fixed	$F_{23} (x) = - \sum\limits_{i = 1}^{7} {[(x - a_{i} )(x - a_{i} )^{T} + c_{i} ]^{ - 1} }$	4	[0,10]	−10.4028
Fixed	$F_{23} (x) = - \sum\limits_{i = 1}^{10} {[(x - a_{i} )(x - a_{i} )^{T} + c_{i} ]^{ - 1} }$	4	[0,10]	−10.5363
Fixed	$F_{24} (x) = x_{1}^{2} + x_{2}^{2} + 25\left( {\sin^{2} (x_{1} ) + \sin^{2} (x_{2} )} \right)$	2	[−5, 5]	0

#### Parameter settings

For fair comparison, seven existing persuasive algorithms are introduced to compare with MSCA, including GA^40^, PSO^11^, GSA^8^, JAYA^41^, ALO^42^, MVO^43^, GWO^44^, WOA^4^, SSA^45^, HGSO^12^, AOA^46^ and SCA^2^. the population size was set to 50, and the maximum iterations was set to 500 in the selected algorithm. For avoid the influence of randomness, each algorithm was independently repeated for 20 times. The default parameter value settings of all the algorithms are listed in Table 2.

**Table 2:** The default parameters of selected algorithm.

Algorithm	Detailed parameter	Value
GA	Mutation probability	0.05
	Crossover probability	0.6
PSO	Cognitive coefficient(*C*_1_)	2.0
	Cognitive coefficient(*C*_2_)	2.0
	Weight (*w*_max_)	0.8
	Weight (*w*_min_)	0.3
GSA	Attenuation factor *a*	20
	Initial gravitational constant *G*_0_	100
MVO	Minimum of Wormhole Existence Probability	0.2
	Maximum of Wormhole Existence Probability	1.0
GWO	Constant *a*	2.0
WOA	Constant *a*	2.0
SSA	Coefficient *c*_1_	$2\exp \left[ {{{\left( { - 4k} \right)} \mathord{\left/ {\vphantom {{\left( { - 4k} \right)} {\overline{k} }}} \right. \kern-\nulldelimiterspace} {\overline{k} }}} \right]^{2}$
HGSO	Cluster number	5
AOA	Control parameter $\mu$	0.5
	Control parameter $\alpha$	5
SCA	Constant *a*	2.0
MSCA	Constant *a*	2.0
	Tuning parameter *β*	1.5

### Experimental results analysis

To obey the univariate principle, all algorithms are run independently in the same environment. The comparison results of selected algorithms for 24 benchmark problems are given in Table 3, including the mean and standard deviation (STD). The results of the Wilcoxon ranksum and signed-rank test at 5% level significance level are reported in Tables 4 and 5, respectively. It should be pointed out that in Table 5 , if the result of MSCA is better than the competitive algorithms, that the MSCA is recorded as win, if equal, recorded as tie, otherwise recorded as lose.

**Table 3:** Comparison results of selected algorithms for classical benchmark problem.

Function	Item	GA	PSO	GSA	JAYA	ALO	MVO	GWO	WOA	SSA	HGSO	AOA	SCA	MSCA
*F*_1_	Mean	1.45E + 03	8.84E-04	4.85E-12	3.98E + 01	1.43E-04	8.62E-01	1.93E-19	1.91E-69	2.85E-05	3.02E-17	3.51E-23	4.72E + 00	**1.13E-75**
	STD	2.51E + 02	9.82E-04	6.97E-12	9.40E + 00	7.88E-05	2.07E-01	2.63E-19	7.12E-69	5.78E-05	1.14E-16	1.57E-22	8.71E + 00	**6.21E-75**
*F*_2_	Mean	1.28E + 01	5.00E + 00	7.37E-02	6.14E + 00	4.80E + 01	1.12E + 01	4.98E-12	8.59E-52	3.25E + 00	1.58E-15	**3.26E-115**	1.38E-02	6.76E-47
	STD	1.71E + 00	6.82E + 00	2.64E-01	2.54E + 00	4.86E + 01	3.22E + 01	2.95E-12	3.72E-51	1.91E + 00	5.42E-15	**1.01E-114**	2.05E-02	1.94E-46
*F*_3_	Mean	2.07E + 04	5.90E + 03	7.04E + 02	3.85E + 04	2.35E + 03	7.84E + 01	5.07E-03	5.96E + 04	2.25E + 03	2.04E + 02	**5.01E-03**	6.92E + 03	8.51E + 00
	STD	4.07E + 03	4.76E + 03	2.22E + 02	5.66E + 03	1.13E + 03	3.74E + 01	1.38E-02	1.91E + 04	7.83E + 02	3.76E + 02	**1.12E-02**	4.67E + 03	1.48E + 01
*F*_4_	Mean	2.26E + 01	7.77E + 00	4.03E + 00	2.74E + 01	1.75E + 01	1.47E + 00	1.92E-04	4.51E + 01	1.54E + 01	9.38E-02	3.51E-02	2.46E + 01	**2.18E-32**
	STD	9.68E-01	1.77E + 00	1.14E + 00	5.56E + 00	5.31E + 00	6.18E-01	1.64E-04	2.87E + 01	5.75E + 00	1.94E-01	1.31E-02	9.57E + 00	**1.19E-31**
*F*_5_	Mean	2.56E + 05	3.85E + 03	8.91E + 01	3.88E + 03	2.53E + 02	3.62E + 02	2.79E + 01	2.85E + 01	4.50E + 02	2.82E + 01	2.85E + 01	1.61E + 04	**2.55E + 01**
	STD	6.47E + 04	1.63E + 04	6.94E + 01	2.18E + 03	3.73E + 02	5.68E + 02	7.92E-01	3.41E-01	7.22E + 02	2.86E-01	2.40E-01	4.24E + 04	**1.66E-01**
*F*_6_	Mean	1.41E + 03	1.03E-03	**6.09E-12**	6.79E + 01	1.04E-04	8.26E-01	1.95E + 00	1.09E + 00	1.74E-05	3.40E + 00	3.84E + 00	8.48E + 00	8.46E-04
	STD	2.46E + 02	1.10E-03	**1.05E-11**	2.22E + 01	5.34E-05	2.07E-01	6.30E-01	3.43E-01	2.26E-05	3.39E-01	2.60E-01	5.47E + 00	1.99E-03
*F*_7_	Mean	6.54E-01	1.04E + 00	5.55E-01	7.09E-01	5.72E-01	5.25E-01	**3.67E-01**	5.05E-01	6.95E-01	5.13E-01	4.54E-01	6.49E-01	4.52E-01
	STD	3.14E-01	2.13E + 00	2.95E-01	2.85E-01	2.67E-01	2.74E-01	3.19E-01	**2.21E-01**	3.47E-01	2.84E-01	2.33E-01	2.57E-01	2.98E-01
*F*_8_	Mean	-1.13E + 04	-9.78E + 03	-2.87E + 03	-5.19E + 03	-5.78E + 03	-8.13E + 03	-5.45E + 03	-9.38E + 03	-7.23E + 03	-4.07E + 03	**-3.03E + 38**	-3.90E + 03	-1.24E + 04
	STD	**2.02E + 02**	6.03E + 02	4.19E + 02	5.98E + 02	8.90E + 02	7.61E + 02	8.05E + 02	1.77E + 03	8.03E + 02	5.81E + 02	1.35E + 39	2.99E + 02	6.26E + 02
*F*_9_	Mean	5.55E + 01	5.72E + 01	1.87E + 01	2.56E + 02	8.34E + 01	1.12E + 02	1.74E + 01	**0.00E + 00**	5.16E + 01	8.27E + 00	**0.00E + 00**	3.90E + 01	**0.00E + 00**
	STD	6.38E + 00	1.81E + 01	4.11E + 00	2.00E + 01	2.03E + 01	3.14E + 01	5.25E + 00	**0.00E + 00**	1.43E + 01	2.69E + 01	**0.00E + 00**	3.87E + 01	**0.00E + 00**
*F*_10_	Mean	8.93E + 00	2.44E-01	9.00E-07	7.70E + 00	2.38E + 00	1.51E + 00	9.54E-11	4.09E-15	3.57E + 00	5.34E-10	**0.00E + 00**	1.38E + 01	**0.00E + 00**
	STD	6.41E-01	4.66E-01	4.97E-07	6.11E + 00	8.25E-01	6.08E-01	6.30E-11	2.89E-15	1.18E + 00	1.82E-09	**0.00E + 00**	9.08E + 00	**0.00E + 00**
*F*_11_	Mean	1.40E + 01	1.72E-02	1.70E + 01	1.39E + 00	2.28E-02	7.58E-01	8.04E-03	**0.00E + 00**	4.53E-02	4.53E-12	3.47E-01	8.24E-01	**0.00E + 00**
	STD	2.11E + 00	1.56E-02	3.87E + 00	1.11E-01	1.27E-02	9.93E-02	1.47E-02	**0.00E + 00**	2.88E-02	1.96E-11	1.82E-01	3.17E-01	**0.00E + 00**
*F*_12_	Mean	9.83E + 01	2.84E-01	1.18E + 00	1.93E + 01	1.15E + 01	1.35E + 00	1.55E-01	7.90E-02	8.39E + 00	5.33E-01	6.18E-01	4.07E + 03	**2.42E-05**
	STD	1.85E + 02	3.95E-01	7.00E-01	9.80E + 00	4.75E + 00	1.10E + 00	1.32E-01	3.32E-02	3.15E + 00	1.50E-01	6.88E-02	2.21E + 04	**5.04E-06**
*F*_13_	Mean	7.12E + 04	5.61E-02	8.22E + 00	4.26E + 01	1.17E + 01	1.21E-01	1.40E + 00	1.28E + 00	2.86E + 01	2.48E + 00	2.67E + 00	3.81E + 04	**3.78E-03**
	STD	3.87E + 04	7.19E-02	6.16E + 00	1.01E + 02	1.47E + 01	4.92E-02	2.78E-01	3.61E-01	1.40E + 01	1.77E-01	1.24E-01	1.91E + 05	**1.87E-02**
*F*_14_	Mean	**9.98E-01**	**9.98E-01**	4.03E + 00	**9.98E-01**	1.63E + 00	**9.98E-01**	5.93E + 00	3.20E + 00	1.25E + 00	1.38E + 00	8.37E + 00	1.40E + 00	**9.98E-01**
	STD	9.96E-09	**4.12E-17**	2.98E + 00	2.73E-04	8.82E-01	5.00E-11	4.81E + 00	3.66E + 00	4.42E-01	4.71E-01	4.53E + 00	8.07E-01	1.43E-15
*F*_15_	Mean	1.05E-03	2.77E-03	6.28E-03	6.32E-04	3.50E-03	5.42E-03	4.41E-03	6.49E-04	4.81E-03	**5.46E-04**	1.88E-02	9.99E-04	6.13E-04
	STD	4.01E-04	5.97E-03	3.33E-03	4.37E-04	6.73E-03	8.39E-03	8.19E-03	3.83E-04	8.04E-03	**1.30E-04**	3.39E-02	3.36E-04	4.11E-04
*F*_16_	Mean	**-1.03E + 00**	**-1.03E + 00**	**-1.03E + 00**	**-1.03E + 00**	**-1.03E + 00**	**-1.03E + 00**	**-1.03E + 00**	**-1.03E + 00**	**-1.03E + 00**	**-1.03E + 00**	**-1.03E + 00**	**-1.03E + 00**	**-1.03E + 00**
	STD	2.27E-05	**6.71E-16**	1.00E-15	3.05E-05	7.51E-14	5.14E-07	1.26E-08	6.85E-09	6.21E-14	1.80E-05	1.89E-07	3.75E-05	1.58E-13
*F*_17_	Mean	**3.98E-01**	**3.98E-01**	**3.98E-01**	3.99E-01	**3.98E-01**	**3.98E-01**	**3.98E-01**	**3.98E-01**	**3.98E-01**	3.99E-01	4.18E-01	4.00E-01	**3.98E-01**
	STD	8.57E-05	**0.00E + 00**	8.97E-15	1.66E-03	3.89E-14	7.72E-07	2.13E-06	3.99E-04	6.10E-13	1.37E-03	1.27E-02	1.67E-03	7.78E-11
*F*_18_	Mean	**3.00E + 00**	**3.00E + 00**	**3.00E + 00**	**3.00E + 00**	**3.00E + 00**	**3.00E + 00**	**3.00E + 00**	**3.00E + 00**	**3.00E + 00**	**3.00E + 00**	2.17E + 01	**3.00E + 00**	**3.00E + 00**
	STD	4.14E-04	**1.24E-15**	1.10E-13	7.97E-04	3.65E-13	4.09E-06	1.53E-04	3.99E-03	8.44E-13	3.75E-04	3.20E + 01	2.47E-05	7.71E-13
*F*_19_	Mean	**-3.86E + 00**	**-3.86E + 00**	-3.70E + 00	**-3.86E + 00**	**-3.86E + 00**	**-3.86E + 00**	**-3.86E + 00**	-3.83E + 00	**-3.86E + 00**	**-3.86E + 00**	-3.85E + 00	**-3.86E + 00**	**-3.86E + 00**
	STD	7.35E-06	**2.65E-15**	2.46E-01	2.71E-15	1.00E-13	2.19E-06	2.09E-03	5.36E-02	2.39E-02	3.36E-03	4.64E-03	3.40E-03	9.53E-10
*F*_20_	Mean	-3.28E + 00	-3.28E + 00	-2.29E + 00	-3.24E + 00	-3.26E + 00	-3.25E + 00	-3.27E + 00	-3.08E + 00	-3.28E + 00	-3.27E + 00	-3.00E + 00	-3.03E + 00	**-3.29E + 00**
	STD	5.69E-02	6.57E-02	7.37E-01	6.01E-02	6.06E-02	5.89E-02	8.36E-02	2.68E-01	8.77E-02	6.66E-02	1.06E-01	1.37E-01	**5.11E-02**
*F*_21_	Mean	-6.95E + 00	-8.48E + 00	-4.98E + 00	-7.22E + 00	-5.94E + 00	-6.87E + 00	**-9.53E + 00**	-7.52E + 00	-9.40E + 00	-8.09E + 00	-4.40E + 00	-2.56E + 00	-9.30E + 00
	STD	3.54E + 00	2.90E + 00	**4.22E-01**	2.46E + 00	2.75E + 00	3.04E + 00	1.97E + 00	2.54E + 00	1.85E + 00	1.99E + 00	1.74E + 00	1.94E + 00	1.93E + 00
*F*_22_	Mean	-7.45E + 00	-8.28E + 00	-8.10E + 00	-8.99E + 00	-7.25E + 00	-9.80E + 00	-1.01E + 01	-7.80E + 00	-8.96E + 00	-7.49E + 00	-3.62E + 00	-4.34E + 00	**-1.02E + 01**
	STD	3.49E + 00	3.12E + 00	2.68E + 00	2.15E + 00	3.08E + 00	1.88E + 00	1.19E + 00	2.81E + 00	2.60E + 00	2.41E + 00	1.66E + 00	1.62E + 00	**9.70E-01**
*F*_23_	Mean	-7.39E + 00	-9.61E + 00	-9.11E + 00	-8.96E + 00	-8.08E + 00	-9.73E + 00	-9.72E + 00	-7.09E + 00	**-1.05E + 01**	-8.09E + 00	-3.85E + 00	-4.70E + 00	-9.82E + 00
	STD	3.64E + 00	2.44E + 00	2.70E + 00	2.27E + 00	3.36E + 00	2.14E + 00	2.50E + 00	2.83E + 00	**3.26E-10**	2.29E + 00	1.91E + 00	1.23E + 00	1.87E + 00
*F*_24_	Mean	9.09E-05	8.77E-73	3.38E-16	6.39E-47	2.41E-13	1.90E-06	2.75E-148	8.74E-94	1.15E-13	1.07E-118	**0.00E + 00**	8.00E-78	2.31E-229
	STD	1.13E-04	1.77E-72	6.02E-16	2.08E-46	2.21E-13	1.67E-06	1.21E-147	3.91E-93	1.12E-13	3.92E-118	**0.00E + 00**	2.99E-77	**0.00E + 00**

Significant values are in bold.

**Table 4:** Wilcoxon ranksum test on classical benchmark functions at 5% level of significance.

Function	GA vs MSCA	PSO vs MSCA	GSA vs MSCA	JAYA vs MSCA	ALO vs MSCA	MVO vs MSCA	GWO vs MSCA	WOA vs MSCA	SSA vs MSCA	HGSO vs MSCA	AOA vs MSCA	SCA vs MSCA
*F*_1_	**6.80E-08**	**6.80E-08**	**6.80E-08**	**6.80E-08**	**6.80E-08**	**6.80E-08**	**6.80E-08**	1.92E-07	**6.80E-08**	**6.80E-08**	**6.80E-08**	**6.80E-08**
*F*_2_	**6.80E-08**	**6.80E-08**	**6.80E-08**	**6.80E-08**	**6.80E-08**	**6.80E-08**	**6.80E-08**	3.94E-07	**6.80E-08**	**6.80E-08**	**6.80E-08**	**6.80E-08**
*F*_3_	**6.80E-08**	**6.80E-08**	**6.80E-08**	**6.80E-08**	**6.80E-08**	1.06E-07	3.29E-05	**6.80E-08**	**6.80E-08**	4.90E-01	2.69E-06	**6.80E-08**
*F*_4_	**6.80E-08**	**6.80E-08**	**6.80E-08**	**6.80E-08**	**6.80E-08**	**6.80E-08**	**6.80E-08**	**6.80E-08**	**6.80E-08**	**6.80E-08**	**6.80E-08**	**6.80E-08**
*F*_5_	**6.80E-08**	1.20E-06	**6.80E-08**	**6.80E-08**	1.20E-06	**6.80E-08**	**6.80E-08**	**6.80E-08**	**6.80E-08**	**6.80E-08**	**6.80E-08**	**6.80E-08**
*F*_6_	**6.80E-08**	4.57E-01	**6.80E-08**	**6.80E-08**	**6.80E-08**	**6.80E-08**	**6.80E-08**	**6.80E-08**	**6.80E-08**	**6.80E-08**	**6.80E-08**	**6.80E-08**
*F*_7_	**6.80E-08**	**6.80E-08**	**6.80E-08**	**6.80E-08**	**6.80E-08**	**6.80E-08**	1.16E-04	**6.80E-08**	**6.80E-08**	**6.80E-08**	3.94E-07	2.56E-07
*F*_8_	1.20E-06	6.92E-07	6.80E-08	6.80E-08	**6.46E-08**	1.23E-07	6.80E-08	1.80E-06	6.80E-08	6.80E-08	1.60E-05	6.80E-08
*F*_9_	**8.01E-09**	**8.01E-09**	**8.01E-09**	**8.01E-09**	**8.01E-09**	**8.01E-09**	**8.01E-09**	N/A	**8.01E-09**	6.68E-05	N/A	**8.01E-09**
*F*_10_	8.01E-09	8.01E-09	8.01E-09	8.01E-09	8.01E-09	8.01E-09	8.01E-09	2.71E-06	8.01E-09	**7.86E-09**	N/A	8.01E-09
*F*_11_	**8.01E-09**	**8.01E-09**	**8.01E-09**	**8.01E-09**	**8.01E-09**	**8.01E-09**	3.37E-07	N/A	**8.01E-09**	1.98E-02	**8.01E-09**	**8.01E-09**
*F*_12_	**6.80E-08**	**6.80E-08**	**6.80E-08**	**6.80E-08**	**6.80E-08**	**6.80E-08**	**6.80E-08**	**6.80E-08**	**6.80E-08**	**6.80E-08**	**6.80E-08**	**6.80E-08**
*F*_13_	**6.80E-08**	6.92E-07	7.90E-08	**6.80E-08**	9.17E-08	2.56E-07	**6.80E-08**	**6.80E-08**	**6.80E-08**	**6.80E-08**	**6.80E-08**	**6.80E-08**
*F*_14_	4.67E-08	**4.91E-09**	8.85E-07	4.67E-08	4.41E-01	4.67E-08	4.61E-08	4.67E-08	5.88E-01	4.67E-08	4.67E-08	4.67E-08
*F*_15_	3.15E-02	9.07E-02	**6.80E-08**	1.64E-01	3.15E-02	6.04E-03	9.25E-01	3.51E-01	9.79E-03	3.94E-01	9.21E-04	9.79E-03
*F*_16_	6.66E-08	**7.80E-09**	**7.80E-09**	6.66E-08	1.14E-02	6.66E-08	6.66E-08	6.66E-08	2.48E-03	6.66E-08	6.66E-08	6.66E-08
*F*_17_	6.80E-08	**8.01E-09**	4.63E-08	1.71E-01	8.50E-07	6.80E-08	6.80E-08	6.80E-08	1.41E-05	6.80E-08	6.80E-08	6.80E-08
*F*_18_	6.76E-08	**7.95E-09**	2.26E-04	6.76E-08	4.02E-01	6.76E-08	6.76E-08	6.76E-08	8.29E-01	6.76E-08	8.47E-07	6.76E-08
*F*_19_	6.80E-08	**8.01E-09**	6.80E-08	**8.01E-09**	6.46E-08	6.80E-08	6.80E-08	6.80E-08	9.13E-07	6.80E-08	6.80E-08	6.80E-08
*F*_20_	6.61E-05	2.34E-01	1.61E-04	8.37E-01	9.25E-01	5.87E-06	1.16E-04	2.06E-06	7.11E-03	3.75E-04	**6.80E-08**	2.22E-07
*F*_21_	2.60E-05	2.81E-03	7.94E-04	1.78E-03	9.79E-03	2.60E-05	8.36E-04	1.60E-05	4.17E-05	2.47E-04	5.87E-06	**1.23E-07**
*F*_22_	6.92E-07	2.25E-02	4.39E-01	6.74E-01	4.90E-01	1.05E-06	1.05E-06	3.42E-07	5.12E-03	4.54E-07	**1.23E-07**	1.92E-07
*F*_23_	1.58E-06	6.80E-05	8.00E-05	3.09E-01	7.64E-02	7.58E-06	9.75E-06	6.92E-07	**6.80E-08**	4.54E-06	2.22E-07	1.66E-07
*F*_24_	6.80E-08	6.80E-08	6.80E-08	6.80E-08	6.80E-08	6.80E-08	6.80E-08	6.80E-08	6.80E-08	6.80E-08	**8.01E-09**	6.80E-08

Significant values are in bold.

**Table 5:** Wilcoxon signed-rank test on classical benchmark functions at 5% level of significance.

Item	Win	Tie	Lose	R^+^	R^-^	*p*-value	Decision
GA vs MSCA	24	0	0	300	0	1.82E-05	+
PSO vs MSCA	19	0	5	280	20	2.04E-04	+
GSA vs MSCA	20	0	4	283	17	1.45E-04	+
JAYA vs MSCA	23	0	1	298	2	2.35E-05	+
ALO vs MSCA	19	0	5	281	19	1.82E-04	+
MVO vs MSCA	24	0	0	300	0	1.82E-05	+
GWO vs MSCA	21	0	3	248	52	5.11E-03	+
WOA vs MSCA	21	2	1	293.5	6.5	6.08E-05	+
SSA vs MSCA	19	0	5	263	37	1.24E-03	+
AOA vs MSCA	18	2	4	245.5	54.5	8.15E-03	+
SCA vs MSCA	24	0	0	300	0	1.82E-05	+

From Table 3, MSCA outperforms GA, MVO and SCA on the mean of all benchmark problems, and the standard deviation of MSCA is smaller than SCA. Furthermore, MSCA wins most problems compared to PSO, GSA, JAYA and ALO.

In Table 4, The p-value of the vast majority of rank sum tests are less than 5%, which illustrates the variability in the overall distribution of target values for the proposed and control methods. At the same time, MSCA achieved *p*-values of less than 5% in all sign-rank comparisons in Table 5, indicating that the results obtained by MSCA are statistically significant. Overall, MSCA can balance the process between exploration and exploitation.

#### Robustness and convergence analysis

**Figure 5:**
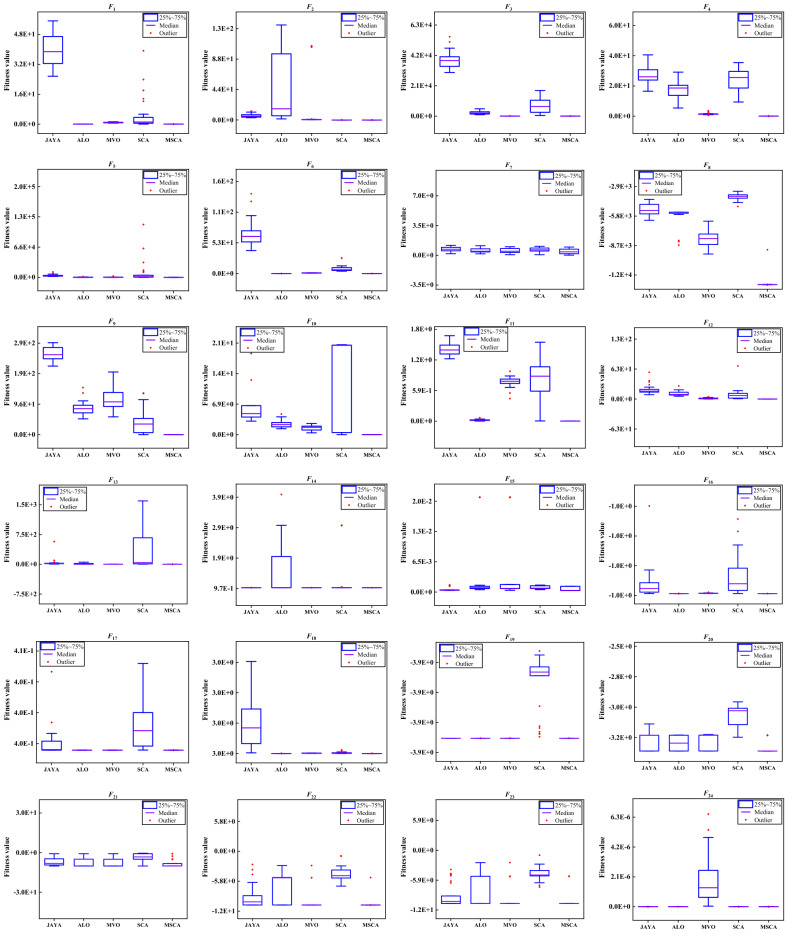
The box-plot of different algorithms on classical benchmark problems.

**Figure 6:**
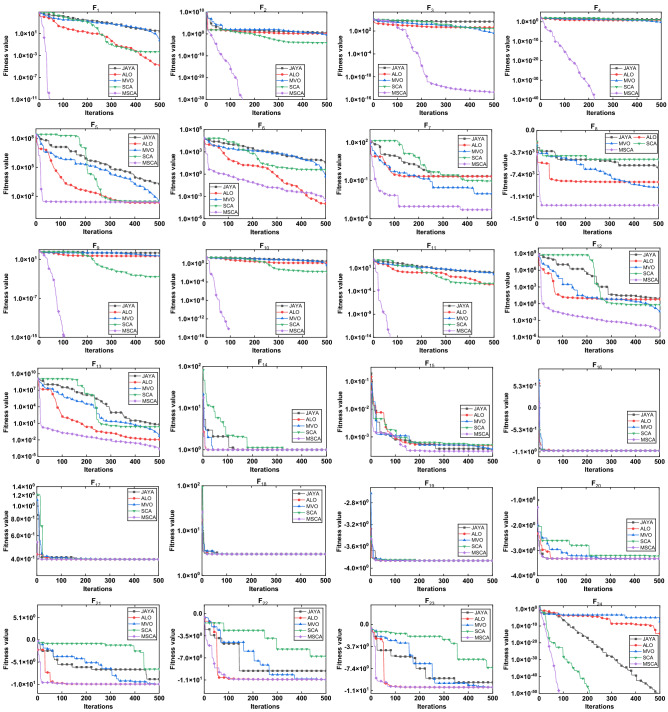
The convergence curve on classical benchmark problems.

For validate the robustness and convergence performance of MSCA in classical benchmark problems, the box-plot of all results and convergence curve of best result for JAYA, ALO, MVO, SCA and MSCA are plotted in Figs. 5 and 6.respectively.

Box-plot revealed the distribution of results obtained by each method. It can be seen from Fig. 5 that JAYA, ALO, MVO and SCA show different degrees of dispersion in different benchmark problems, while MCSA get more stable and better distribution. And it can be seen from the convergence curve in Fig. 6 that MSCA has a faster convergence rate than other algorithms. Hence, the modified SCA position update formula combined with Levy random walk mutation strategy can get more accurate results.

### Benchmark problem set II: CEC2017 test suites

In this section, IEEE CEC2017 test suites are adopted to prove the performance of MSCA, which are mainly divided into four groups: (a) Unimodal, (b) Multimodal, (c) Hybrid and (d) Composition. The definition of the CEC2017 benchmark problems is shown in Table 6. Unlike classical benchmark function problems, this problem involves matrix rotation and global optimum shifted operations.

**Table 6:** The definition of CEC2017 test suites.

Type	Function	Name	*n*	_Range_	*f*_min_
Unimodal	*F*_1_:	Shifted and Rotated Bent Cigar Function	10	[−100, 100]	100
Unimodal	*F*_2_:	Shifted and Rotated Sum of Different Power Function	10	[−100, 100]	200
Unimodal	*F*_3_:	Shifted and Rotated Zakharov Function	10	[−100, 100]	300
Multimodal	*F*_4_:	Shifted and Rotated Rosenbrock’s Function	10	[−100, 100]	400
Multimodal	*F*_5_:	Shifted and Rotated Rastrigin’s Function	10	[−100, 100]	500
Multimodal	*F*_6_:	Shifted and Rotated Expanded Scaffer’s *F*_6_ Function	10	[−100, 100]	600
Multimodal	*F*_7_:	Shifted and Rotated Lunacek Bi_Rastrigin Function	10	[−100, 100]	700
Multimodal	*F*_8_:	Shifted and Rotated Non−Continuous Rastrigin’s Function	10	[−100, 100]	800
Multimodal	*F*_9_:	Shifted and Rotated Levy Function	10	[−100, 100]	900
Multimodal	*F*_10_:	Shifted and Rotated Schwefel’s Function	10	[−100, 100]	1000
Hybrid	*F*_11_:	Hybrid Function 1 (*N* = 3)	10	[−100, 100]	1100
Hybrid	*F*_12_:	Hybrid Function 2 (*N* = 3)	10	[−100, 100]	1200
Hybrid	*F*_13_:	Hybrid Function 3 (*N* = 3)	10	[−100, 100]	1300
Hybrid	*F*_14_:	Hybrid Function 4 (*N* = 4)	10	[−100, 100]	1400
Hybrid	*F*_15_:	Hybrid Function 5 (*N* = 4)	10	[−100, 100]	1500
Hybrid	*F*_16_:	Hybrid Function 6 (*N* = 4)	10	[−100, 100]	1600
Hybrid	*F*_17_:	Hybrid Function 6 (*N* = 5)	10	[−100, 100]	1700
Hybrid	*F*_18_:	Hybrid Function 6 (*N* = 5)	10	[−100, 100]	1800
Hybrid	*F*_19_:	Hybrid Function 6 (*N* = 5)	10	[−100, 100]	1900
Hybrid	*F*_20_:	Hybrid Function 6 (*N* = 6)	10	[−100, 100]	2000
Composition	*F*_21_:	Composition Function 1 (*N* = 3)	10	[−100, 100]	2100
Composition	*F*_22_:	Composition Function 2 (*N* = 3)	10	[−100, 100]	2200
Composition	*F*_23_:	Composition Function 3 (*N* = 4)	10	[−100, 100]	2300
Composition	*F*_24_:	Composition Function 4 (*N* = 4)	10	[−100, 100]	2400
Composition	*F*_25_:	Composition Function 5 (*N* = 5)	10	[−100, 100]	2500
Composition	*F*_26_:	Composition Function 6 (*N* = 5)	10	[−100, 100]	2600
Composition	*F*_27_:	Composition Function 7 (*N* = 6)	10	[−100, 100]	2700
Composition	*F*_28_:	Composition Function 8 (*N* = 6)	10	[−100, 100]	2800
Composition	*F*_29_:	Composition Function 9 (*N* = 3)	10	[−100, 100]	2900
Composition	*F*_30_:	Composition Function 10 (*N* = 3)	10	[−100, 100]	3000

#### Experimental results analysis

The statistical results for CEC2017 are illustrated in Table 7. From the table, it can be concluded that MSCA outperforms the other popular methods in most of the CEC2017 benchmark problems. In addition, from the results of the Wilcoxon ranksum and signed-rank test in Tables 8 and 9, it can be concluded that the proposed method is effective in improving the search efficiency of SCA and provides better results than other methods. In conclusion, MSCA is a quite competitive algorithm.

**Table 7:** Statistic result of the algorithms on CEC2017 test suit with 10 dimensions.

Function	Item	GA	PSO	GSA	JAYA	ALO	MVO	GWO	WOA	SSA	HGSO	AOA	SCA	MSCA
*F*_1_	Mean	1.12E + 07	9.35E + 06	**3.28E + 02**	3.81E + 08	1.84E + 03	1.69E + 04	1.78E + 08	4.47E + 08	2.13E + 03	4.40E + 08	1.02E + 10	8.37E + 08	1.55E + 05
	STD	5.63E + 06	2.42E + 07	**4.24E + 02**	1.49E + 08	1.89E + 03	5.93E + 03	2.06E + 08	2.87E + 08	2.22E + 03	4.52E + 08	4.29E + 09	2.45E + 08	8.44E + 04
*F*_2_	Mean	6.44E + 06	1.04E + 09	4.60E + 10	4.31E + 07	1.43E + 03	**7.26E + 02**	5.45E + 09	5.69E + 09	2.04E + 05	6.10E + 08	8.30E + 14	1.19E + 09	1.74E + 05
	STD	1.22E + 07	5.58E + 09	7.19E + 10	4.05E + 07	2.69E + 03	**1.03E + 03**	9.60E + 09	1.58E + 10	3.55E + 05	1.86E + 09	1.39E + 15	2.48E + 09	3.18E + 05
*F*_3_	Mean	3.79E + 03	**3.00E + 02**	1.58E + 04	8.46E + 03	**3.00E + 02**	**3.00E + 02**	5.17E + 03	1.24E + 04	3.45E + 02	3.28E + 03	1.50E + 04	2.14E + 03	3.79E + 02
	STD	1.47E + 03	**3.60E-03**	3.32E + 03	2.05E + 03	1.68E-01	7.99E-02	2.85E + 03	8.10E + 03	1.36E + 02	1.30E + 03	4.11E + 03	1.35E + 03	5.77E + 01
*F*_4_	Mean	4.07E + 02	4.24E + 02	4.25E + 02	4.10E + 02	4.11E + 02	**4.05E + 02**	4.29E + 02	4.80E + 02	4.06E + 02	4.35E + 02	1.13E + 03	4.54E + 02	4.07E + 02
	STD	9.05E-01	3.94E + 01	1.79E + 01	6.11E-01	1.76E + 01	1.47E + 00	2.82E + 01	5.93E + 01	2.20E + 00	1.82E + 01	4.70E + 02	2.06E + 01	**5.94E-01**
*F*_5_	Mean	5.68E + 02	1.08E + 03	6.13E + 02	1.13E + 03	**5.24E + 02**	5.29E + 02	5.25E + 02	5.69E + 02	5.30E + 02	5.44E + 02	5.71E + 02	1.95E + 03	5.34E + 02
	STD	1.42E + 01	8.77E + 02	1.73E + 02	2.05E + 02	8.60E + 00	1.19E + 01	1.37E + 01	2.17E + 01	1.32E + 01	8.66E + 00	1.89E + 01	4.93E + 02	**6.38E + 00**
*F*_6_	Mean	**6.00E + 02**	**6.00E + 02**	**6.00E + 02**	**6.00E + 02**	**6.00E + 02**	**6.00E + 02**	6.05E + 02	6.45E + 02	6.21E + 02	6.14E + 02	6.42E + 02	**6.00E + 02**	**6.00E + 02**
	STD	3.47E-03	4.87E-03	3.24E-02	1.10E-02	2.79E-02	1.13E-02	3.49E + 00	1.33E + 01	1.05E + 01	7.71E + 00	5.27E + 00	1.58E-02	**1.51E-04**
*F*_7_	Mean	7.37E + 02	**7.24E + 02**	**7.24E + 02**	7.63E + 02	7.41E + 02	7.29E + 02	7.41E + 02	7.94E + 02	7.39E + 02	7.65E + 02	7.93E + 02	7.81E + 02	7.29E + 02
	STD	5.81E + 00	5.27E + 00	5.67E + 00	8.03E + 00	1.41E + 01	9.68E + 00	1.57E + 01	2.01E + 01	1.16E + 01	1.17E + 01	1.27E + 01	1.01E + 01	**4.03E + 00**
*F*_8_	Mean	8.16E + 02	8.13E + 02	8.23E + 02	8.47E + 02	8.21E + 02	8.19E + 02	8.20E + 02	8.47E + 02	8.30E + 02	8.40E + 02	8.45E + 02	8.45E + 02	**8.09E + 02**
	STD	3.26E + 00	5.14E + 00	6.23E + 00	6.18E + 00	1.03E + 01	9.59E + 00	7.64E + 00	1.84E + 01	9.80E + 00	7.50E + 00	1.25E + 01	7.41E + 00	**2.69E + 00**
*F*_9_	Mean	**9.00E + 02**	**9.00E + 02**	**9.00E + 02**	**9.00E + 02**	**9.00E + 02**	**9.00E + 02**	9.46E + 02	1.81E + 03	1.03E + 03	9.79E + 02	1.39E + 03	**9.00E + 02**	**9.00E + 02**
	STD	1.93E-02	1.45E-01	5.83E-01	1.00E-01	3.14E-01	6.88E-01	6.89E + 01	6.45E + 02	1.50E + 02	5.27E + 01	2.13E + 02	1.44E-01	**5.26E-05**
*F*_10_	Mean	1.49E + 03	1.52E + 03	2.90E + 03	2.25E + 03	2.00E + 03	1.70E + 03	1.88E + 03	2.32E + 03	1.96E + 03	2.59E + 03	2.43E + 03	2.40E + 03	**1.29E + 03**
	STD	1.51E + 02	2.15E + 02	2.40E + 02	2.26E + 02	3.94E + 02	2.66E + 02	4.13E + 02	3.71E + 02	3.27E + 02	2.07E + 02	2.66E + 02	2.01E + 02	**1.36E + 02**
*F*_11_	Mean	1.27E + 03	1.17E + 03	1.17E + 04	2.61E + 03	1.17E + 03	1.14E + 03	1.18E + 03	1.36E + 03	1.17E + 03	1.18E + 03	5.60E + 03	2.68E + 03	**1.13E + 03**
	STD	8.15E + 01	3.13E + 02	2.65E + 03	4.85E + 02	6.92E + 01	7.33E + 01	9.02E + 01	2.60E + 02	4.89E + 01	2.79E + 01	6.71E + 03	1.14E + 03	**2.14E + 01**
*F*_12_	Mean	6.64E + 05	1.11E + 06	1.50E + 06	9.96E + 06	1.97E + 06	1.27E + 06	8.08E + 05	4.53E + 06	3.27E + 06	7.92E + 06	3.71E + 08	1.72E + 07	**1.84E + 05**
	STD	5.07E + 05	2.83E + 06	8.49E + 05	9.25E + 06	2.11E + 06	1.13E + 06	8.92E + 05	5.44E + 06	4.58E + 06	6.79E + 06	3.07E + 08	1.68E + 07	**3.41E + 05**
*F*_13_	Mean	1.22E + 04	8.39E + 03	1.13E + 04	1.85E + 04	1.51E + 04	1.30E + 04	1.35E + 04	1.77E + 04	1.66E + 04	3.16E + 04	2.51E + 07	4.33E + 04	**3.97E + 03**
	STD	8.41E + 03	8.48E + 03	**2.18E + 03**	1.63E + 04	1.30E + 04	1.07E + 04	9.55E + 03	1.91E + 04	1.10E + 04	2.71E + 04	6.38E + 07	2.47E + 04	4.03E + 03
*F*_14_	Mean	2.34E + 03	2.08E + 03	1.42E + 04	4.40E + 03	1.24E + 04	1.98E + 03	5.24E + 03	2.97E + 03	4.13E + 03	2.77E + 03	7.80E + 03	6.49E + 03	**1.46E + 03**
	STD	1.03E + 03	1.03E + 03	2.86E + 03	1.70E + 03	6.85E + 03	8.71E + 02	2.40E + 03	1.75E + 03	4.15E + 03	1.35E + 03	7.29E + 03	2.24E + 03	**1.46E + 01**
*F*_15_	Mean	2.95E + 03	4.63E + 03	2.87E + 04	1.02E + 04	3.08E + 04	3.01E + 03	9.88E + 03	1.00E + 04	9.93E + 03	4.50E + 03	1.61E + 04	8.43E + 03	**1.59E + 03**
	STD	1.37E + 03	6.30E + 03	5.87E + 03	6.34E + 03	1.65E + 04	2.27E + 03	6.75E + 03	6.62E + 03	7.74E + 03	2.42E + 03	7.72E + 03	3.37E + 03	**1.32E + 02**
*F*_16_	Mean	1.66E + 03	1.74E + 03	2.25E + 03	1.73E + 03	1.90E + 03	1.75E + 03	1.79E + 03	2.05E + 03	1.82E + 03	1.80E + 03	2.13E + 03	1.80E + 03	**1.62E + 03**
	STD	6.33E + 01	1.34E + 02	1.05E + 02	4.75E + 01	1.85E + 02	1.25E + 02	1.29E + 02	2.08E + 02	1.62E + 02	8.73E + 01	1.81E + 02	9.61E + 01	**3.22E + 01**
*F*_17_	Mean	1.74E + 03	1.78E + 03	1.88E + 03	1.93E + 03	1.83E + 03	1.82E + 03	1.78E + 03	1.84E + 03	1.78E + 03	1.78E + 03	1.89E + 03	1.90E + 03	**1.73E + 03**
	STD	1.93E + 01	6.23E + 01	1.51E + 02	5.63E + 01	1.03E + 02	7.46E + 01	3.27E + 01	6.59E + 01	2.39E + 01	1.25E + 01	8.68E + 01	7.55E + 01	**8.08E + 00**
*F*_18_	Mean	1.40E + 04	3.36E + 04	2.44E + 04	1.01E + 05	4.40E + 04	2.07E + 04	2.14E + 04	1.90E + 04	2.13E + 04	2.44E + 05	2.54E + 08	2.29E + 05	**8.86E + 03**
	STD	1.03E + 04	2.16E + 04	6.49E + 03	5.95E + 04	2.99E + 04	1.44E + 04	1.73E + 04	1.32E + 04	1.44E + 04	3.60E + 05	4.88E + 08	2.43E + 05	**6.47E + 03**
*F*_19_	Mean	5.01E + 03	1.36E + 04	6.44E + 05	1.10E + 04	2.58E + 04	2.47E + 03	2.94E + 04	4.73E + 05	7.15E + 03	6.58E + 03	4.99E + 06	1.24E + 04	**1.97E + 03**
	STD	3.61E + 03	1.65E + 04	5.57E + 05	1.01E + 04	1.77E + 04	8.24E + 02	6.97E + 04	8.96E + 05	6.74E + 03	4.53E + 03	2.15E + 07	8.02E + 03	**1.06E + 02**
*F*_20_	Mean	2.03E + 03	2.08E + 03	2.39E + 03	2.27E + 03	2.20E + 03	2.16E + 03	2.14E + 03	2.24E + 03	2.13E + 03	2.11E + 03	2.16E + 03	2.29E + 03	**2.02E + 03**
	STD	**8.67E + 00**	8.39E + 01	1.34E + 02	6.44E + 01	1.02E + 02	8.87E + 01	7.87E + 01	8.93E + 01	6.71E + 01	3.51E + 01	6.19E + 01	6.56E + 01	2.21E + 01
*F*_21_	Mean	2.27E + 03	2.56E + 03	2.32E + 03	2.67E + 03	2.21E + 03	2.25E + 03	2.32E + 03	2.34E + 03	2.28E + 03	2.24E + 03	2.36E + 03	2.62E + 03	**2.20E + 03**
	STD	5.06E + 01	2.47E + 02	9.78E + 01	6.89E + 01	3.19E + 01	6.49E + 01	9.00E + 00	4.81E + 01	5.97E + 01	2.01E + 01	3.66E + 01	2.15E + 02	**5.03E-01**
*F*_22_	Mean	2.31E + 03	2.32E + 03	2.39E + 03	2.33E + 03	**2.30E + 03**	2.37E + 03	2.37E + 03	2.63E + 03	**2.30E + 03**	2.34E + 03	3.16E + 03	2.39E + 03	2.31E + 03
	STD	1.60E + 00	2.20E + 01	3.40E + 02	4.46E + 00	2.72E + 00	2.45E + 02	1.72E + 02	5.83E + 02	1.69E + 01	3.46E + 01	3.91E + 02	2.86E + 01	**8.96E-01**
*F*_23_	Mean	2.53E + 03	3.00E + 03	4.76E + 03	2.92E + 03	2.42E + 03	**2.41E + 03**	2.63E + 03	2.68E + 03	2.62E + 03	2.66E + 03	2.75E + 03	3.30E + 03	**2.41E + 03**
	STD	3.16E + 01	3.93E + 02	9.91E + 02	2.28E + 02	1.09E + 02	**1.12E + 00**	1.33E + 01	3.35E + 01	8.74E + 00	2.07E + 01	3.86E + 01	1.51E + 02	2.38E + 00
*F*_24_	Mean	2.73E + 03	3.24E + 03	**2.54E + 03**	4.02E + 03	2.61E + 03	2.61E + 03	2.76E + 03	2.78E + 03	2.75E + 03	2.64E + 03	2.90E + 03	3.85E + 03	2.61E + 03
	STD	3.93E + 01	9.10E + 02	6.33E + 01	3.34E + 02	4.87E + 01	5.81E + 01	1.57E + 01	5.69E + 01	**1.19E + 01**	6.55E + 01	1.05E + 02	2.28E + 02	4.12E + 01
*F*_25_	Mean	2.94E + 03	2.95E + 03	2.95E + 03	2.97E + 03	**2.93E + 03**	**2.93E + 03**	2.95E + 03	3.06E + 03	**2.93E + 03**	2.96E + 03	3.42E + 03	2.97E + 03	2.94E + 03
	STD	1.59E + 01	3.17E + 01	**3.43E + 00**	9.57E + 00	2.18E + 01	2.89E + 01	3.27E + 01	1.59E + 02	2.45E + 01	1.57E + 01	2.31E + 02	2.96E + 01	1.90E + 01
*F*_26_	Mean	4.70E + 03	7.29E + 03	5.65E + 03	7.20E + 03	4.60E + 03	3.73E + 03	3.32E + 03	4.03E + 03	**2.94E + 03**	3.13E + 03	4.20E + 03	8.11E + 03	4.38E + 03
	STD	1.35E + 03	2.36E + 03	1.91E + 02	3.80E + 02	1.54E + 03	1.36E + 03	3.55E + 02	5.70E + 02	2.38E + 02	**1.37E + 02**	3.91E + 02	6.80E + 02	1.54E + 03
*F*_27_	Mean	**3.10E + 03**	3.13E + 03	3.64E + 03	3.11E + 03	3.12E + 03	**3.10E + 03**	3.13E + 03	3.18E + 03	**3.10E + 03**	3.12E + 03	3.61E + 03	3.11E + 03	**3.10E + 03**
	STD	3.45E + 00	3.67E + 01	2.55E + 02	2.29E + 01	2.54E + 01	1.78E + 01	4.32E + 01	5.20E + 01	3.77E + 00	1.71E + 01	2.86E + 02	**3.19E + 00**	1.29E + 01
*F*_28_	Mean	**3.22E + 03**	3.35E + 03	3.55E + 03	3.42E + 03	3.33E + 03	3.33E + 03	3.40E + 03	3.54E + 03	3.35E + 03	3.31E + 03	4.00E + 03	3.31E + 03	3.35E + 03
	STD	8.40E + 01	8.50E + 01	**5.74E + 01**	1.38E + 02	1.28E + 02	1.18E + 02	8.62E + 01	1.74E + 02	1.69E + 02	8.05E + 01	2.50E + 02	8.27E + 01	1.05E + 02
*F*_29_	Mean	3.22E + 03	3.31E + 03	3.95E + 03	3.38E + 03	3.34E + 03	3.21E + 03	3.26E + 03	3.46E + 03	3.22E + 03	3.27E + 03	3.45E + 03	3.54E + 03	**3.19E + 03**
	STD	5.22E + 01	9.69E + 01	3.83E + 02	7.49E + 01	1.14E + 02	4.16E + 01	6.39E + 01	1.52E + 02	6.25E + 01	3.59E + 01	1.48E + 02	1.08E + 02	**2.38E + 01**
*F*_30_	Mean	3.13E + 05	5.32E + 05	3.36E + 06	4.45E + 05	4.56E + 05	4.65E + 05	1.12E + 06	2.53E + 06	1.07E + 06	1.29E + 06	2.31E + 07	1.72E + 06	**3.53E + 04**
	STD	4.61E + 05	5.15E + 05	1.22E + 06	3.77E + 05	7.43E + 05	5.64E + 05	9.98E + 05	2.93E + 06	1.54E + 06	9.50E + 05	4.82E + 07	1.20E + 06	**2.51E + 04**

Significant values are in bold.

**Table 8:** Wilcoxon ranksum test on CEC17 test suit at 5% level of significance.

Function	GA vs MSCA	PSO vs MSCA	GSA vs MSCA	JAYA vs MSCA	ALO vs MSCA	MVO vs MSCA	GWO vs MSCA	WOA vs MSCA	SSA vs MSCA	HGSO vs MSCA	AOA vs MSCA	SCA vs MSCA
*F*_1_	**6.80E-08**	1.56E-04	**6.80E-08**	**6.80E-08**	**6.80E-08**	**6.80E-08**	6.01E-02	**6.80E-08**	**6.80E-08**	**6.80E-08**	**6.80E-08**	**6.80E-08**
*F*_2_	2.22E-07	4.25E-01	**6.80E-08**	1.92E-07	1.63E-03	1.61E-04	2.56E-07	**6.80E-08**	1.72E-01	**6.80E-08**	**6.80E-08**	**6.80E-08**
*F*_3_	**6.80E-08**	**6.80E-08**	**6.80E-08**	**6.80E-08**	**6.80E-08**	**6.80E-08**	**6.80E-08**	**6.80E-08**	2.92E-05	**6.80E-08**	**6.80E-08**	**6.80E-08**
*F*_4_	7.64E-02	1.49E-05	1.10E-05	**6.80E-08**	1.23E-02	9.13E-07	2.00E-04	1.20E-06	4.70E-03	**6.80E-08**	**6.80E-08**	**6.80E-08**
*F*_5_	9.17E-08	3.15E-02	4.90E-01	**6.80E-08**	6.22E-04	9.05E-03	1.78E-03	2.56E-07	1.99E-01	3.75E-04	6.92E-07	**6.80E-08**
*F*_6_	**6.80E-08**	5.98E-01	**6.80E-08**	**6.80E-08**	**6.80E-08**	6.01E-07	**6.80E-08**	**6.80E-08**	**6.80E-08**	**6.80E-08**	**6.80E-08**	**6.80E-08**
*F*_7_	6.61E-05	3.06E-03	3.64E-03	**6.80E-08**	4.32E-03	5.65E-02	2.75E-02	**6.80E-08**	6.22E-04	**6.80E-08**	**6.80E-08**	**6.80E-08**
*F*_8_	3.42E-07	2.80E-03	9.07E-08	**6.80E-08**	4.17E-05	3.05E-04	3.07E-06	**6.80E-08**	7.90E-08	**6.80E-08**	**6.80E-08**	**6.80E-08**
*F*_9_	**6.80E-08**	6.03E-06	9.75E-06	**6.80E-08**	3.15E-02	5.08E-01	**6.80E-08**	**6.80E-08**	1.60E-05	**6.80E-08**	**6.80E-08**	**6.80E-08**
*F*_10_	1.41E-05	7.58E-04	**6.80E-08**	**6.80E-08**	**6.80E-08**	3.94E-07	2.96E-07	**6.80E-08**	**6.80E-08**	**6.80E-08**	**6.80E-08**	**6.80E-08**
*F*_11_	2.96E-07	5.63E-04	**6.80E-08**	**6.80E-08**	1.08E-01	7.35E-01	9.79E-03	3.42E-07	4.60E-04	7.58E-06	**6.80E-08**	**6.80E-08**
*F*_12_	1.25E-05	7.41E-05	5.23E-07	9.17E-08	2.14E-03	8.60E-06	8.35E-03	3.50E-06	6.61E-05	9.17E-08	**6.80E-08**	**6.80E-08**
*F*_13_	2.75E-04	1.79E-02	6.61E-05	8.36E-04	1.95E-03	2.14E-03	5.09E-04	2.75E-04	2.04E-05	**7.95E-07**	2.92E-05	9.13E-07
*F*_14_	**6.80E-08**	4.32E-03	**6.80E-08**	**6.80E-08**	**6.80E-08**	5.25E-05	1.23E-07	**6.80E-08**	**6.80E-08**	**6.80E-08**	7.90E-08	**6.80E-08**
*F*_15_	4.54E-07	1.81E-05	**6.80E-08**	**6.80E-08**	**6.80E-08**	2.36E-06	**6.80E-08**	**6.80E-08**	**6.80E-08**	**6.80E-08**	**6.80E-08**	**6.80E-08**
*F*_16_	7.71E-03	6.22E-04	**6.80E-08**	3.94E-07	1.66E-07	9.75E-06	5.23E-07	1.43E-07	6.01E-07	2.56E-07	7.90E-08	1.66E-07
*F*_17_	1.79E-02	2.34E-03	6.01E-07	**6.80E-08**	4.68E-05	7.58E-06	9.17E-08	**6.80E-08**	1.66E-07	**6.80E-08**	**6.80E-08**	**6.80E-08**
*F*_18_	9.09E-02	3.05E-04	9.75E-06	**6.80E-08**	2.04E-05	1.55E-02	4.11E-02	1.79E-02	1.14E-02	3.94E-07	1.01E-03	**6.80E-08**
*F*_19_	1.23E-07	6.92E-07	**6.80E-08**	7.90E-08	**6.80E-08**	4.32E-03	1.20E-06	**6.80E-08**	1.06E-07	7.90E-08	7.90E-08	**6.80E-08**
*F*_20_	6.61E-05	1.95E-03	**6.80E-08**	**6.80E-08**	1.66E-07	2.22E-07	2.22E-07	9.17E-08	2.56E-07	6.01E-07	1.66E-07	**6.80E-08**
*F*_21_	**6.80E-08**	1.60E-05	1.08E-01	**6.80E-08**	**6.80E-08**	3.10E-01	**6.80E-08**	**6.80E-08**	4.11E-02	**6.80E-08**	**6.80E-08**	**6.80E-08**
*F*_22_	**6.80E-08**	5.98E-01	2.23E-02	**6.80E-08**	1.12E-03	8.36E-04	1.14E-02	1.60E-05	5.25E-05	**6.80E-08**	**6.80E-08**	**6.80E-08**
*F*_23_	**6.80E-08**	7.11E-03	**6.80E-08**	**6.80E-08**	1.20E-06	7.90E-08	**6.80E-08**	**6.80E-08**	**6.80E-08**	**6.80E-08**	**6.80E-08**	**6.80E-08**
*F*_24_	3.42E-07	4.57E-01	4.54E-06	**6.80E-08**	7.58E-04	1.23E-02	1.06E-07	1.05E-06	1.66E-07	3.10E-01	9.17E-08	**6.80E-08**
*F*_25_	2.07E-02	5.07E-01	8.36E-04	6.92E-07	3.79E-01	6.56E-03	3.79E-01	6.92E-07	5.61E-01	8.29E-05	**6.80E-08**	5.87E-06
*F*_26_	6.56E-03	9.75E-06	5.98E-01	**6.80E-08**	2.73E-01	5.25E-05	8.60E-01	5.98E-01	1.58E-06	8.82E-01	5.98E-01	**6.80E-08**
*F*_27_	1.23E-03	1.25E-05	**6.80E-08**	4.17E-05	5.90E-05	4.57E-01	7.11E-03	5.23E-07	3.51E-01	1.10E-05	**6.80E-08**	5.25E-05
*F*_28_	9.28E-05	1.25E-01	**6.80E-08**	4.11E-02	4.57E-01	3.38E-04	3.85E-02	8.29E-05	8.39E-01	1.29E-04	9.75E-06	6.39E-02
*F*_29_	1.67E-02	4.70E-03	**6.80E-08**	7.90E-08	1.79E-04	8.82E-01	3.06E-03	**6.80E-08**	2.18E-01	9.13E-07	3.42E-07	**6.80E-08**
*F*_30_	1.44E-04	3.96E-03	**6.80E-08**	1.80E-06	3.64E-03	4.70E-03	1.80E-06	2.92E-05	2.14E-03	**6.80E-08**	**6.80E-08**	7.90E-08

Significant values are in bold.

**Table 9:** Wilcoxon signed-rank test on CEC17 test suit at 5% level of significance.

Item	Win	Tie	Lose	R^+^	R^-^	*p*-value	Decision
GA vs MSCA	29	0	1	448	17	9.32E-06	+
PSO vs MSCA	27	0	3	443	22	1.49E-05	+
GSA vs MSCA	27	0	3	429	36	5.31E-05	+
JAYA vs MSCA	30	0	0	465	0	1.73E-06	+
ALO vs MSCA	23	0	7	364	101	6.84E-03	+
MVO vs MSCA	19	0	11	322	143	6.56E-02	≈
GWO vs MSCA	28	0	2	443	22	1.49E-05	+
WOA vs MSCA	29	0	1	448	17	9.32E-06	+
SSA vs MSCA	21	0	9	384	81	1.83E-03	+
HGSO vs MSCA	28	0	2	435	30	3.11E-05	+
AOA vs MSCA	29	0	1	457	8	3.88E-06	+
SCA vs MSCA	29	0	1	459	6	3.18E-06	+

#### Robustness and convergence analysis

**Figure 7:**
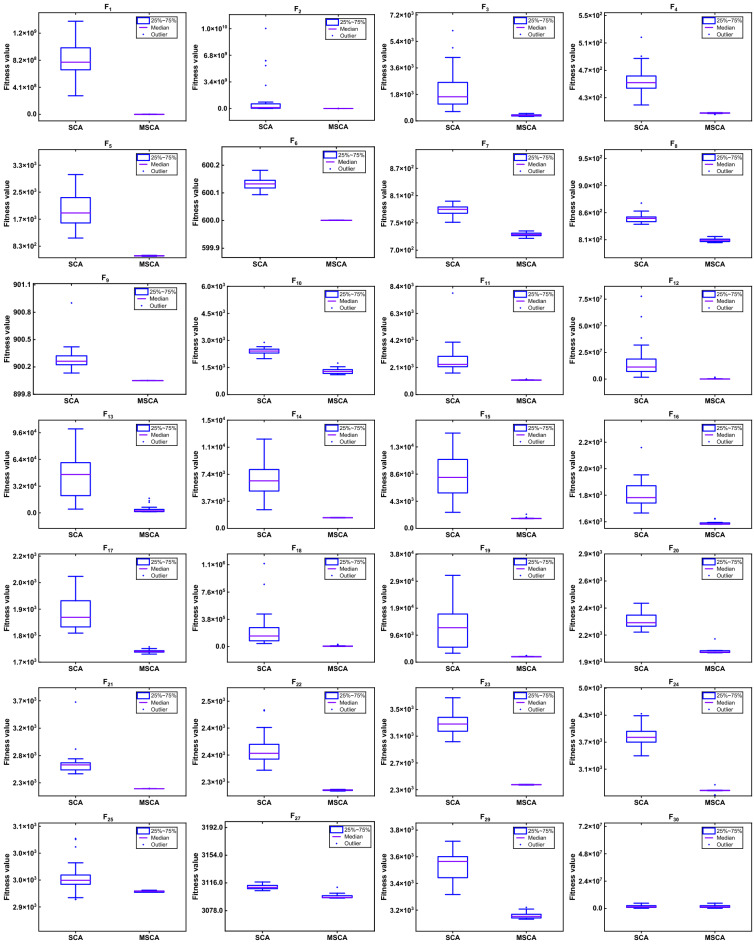
The box-plot of SCA and MSCA on CEC2017.

**Figure 8:**
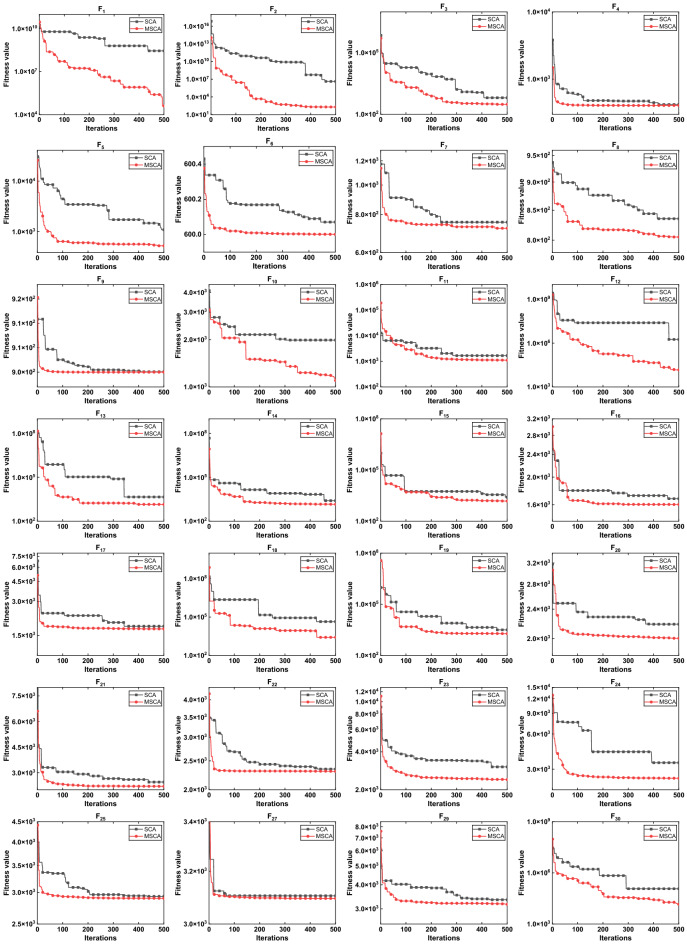
The convergence curve of SCA and MSCA on CEC2017.

The box-plot for MSCA and SCA for CEC2017 are illustrated in Figs. 7 and 8, respectively. From Fig. 7, the overall distribution of the MSCA optimal solution is more concentrated and better. Notably, in Fig. 8, the overall convergence speed of MSCA is faster than that of SCA. On the whole, the proposed algorithm can improve the convergence rate and solution accuracy, which is an effective tool for solving numerical problems.

## MSCA for engineering optimization problems

In this subsection, the proposed MSCA tries to handle six famous constrained engineering problems. The parameters of the MCSA are set the same as in Section “MSCA for numerical optimization problems”. In addition, the static penalty function is employed to handle the equation and inequality constraints involved in the problem. The static penalty function is expressed as follows:

5 $$fit({\varvec{x}}) = f({\varvec{x}}) + \alpha [\sum\nolimits_{i = 1}^{n} {g_{i}^{2} ({\varvec{x}})} + \sum\nolimits_{j = 1}^{p} {\left| {h_{j}^{{}} ({\varvec{x}})} \right|} ]$$

where $fit({\varvec{x}})$ and $f({\varvec{x}})$ are the fitness value and objective value, respectively. $\alpha$ is the penalty coefficient. $n$ is the number of inequality constraints. $g_{i}^{{}} ({\varvec{x}})$ is the degree of violation of the *i*th inequality constraint. $p$ is the number of equality constraints. $h_{j}^{{}} ({\varvec{x}})$ is the degree of violation of the *j*th equality constraint.

### Case I: Three-bar truss design problem

**Figure 9:**
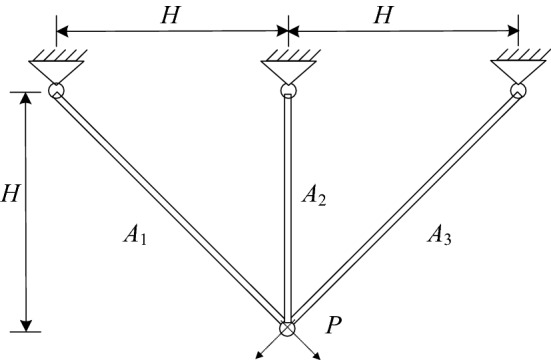
The three-bar truss design.

Trusses belong to one of the prefabricated concrete structures. This problem requires finding the minimum volume of the truss according to the decision variables of cross-sectional areas ${\varvec{x}}$ = $\left( {x_{1} ,x_{2} } \right)$ = $\left( {A_{1} ,A_{2} } \right)$^47^, as plotted in Fig. 9, the problem are described as follows:

6 $$\begin{gathered} {\text{min}}\;\;f({\varvec{x}}) = \left( {2\sqrt 2 x_{1} + x_{2} } \right) \times l \hfill \\ \;\;\;s.t.\;\;g_{1} = \frac{{\sqrt 2 x_{1} + x_{2} }}{{\sqrt 2 x_{1}^{2} + 2x_{1} x_{2} }}P - \sigma \le 0 \hfill \\ \;\;\;\;\;\;\;\;g_{2} = \frac{{x_{2} }}{{\sqrt 2 x_{1}^{2} + 2x_{1} x_{2} }}P - \sigma \le 0 \hfill \\ \;\;\;\;\;\;\;\;g_{3} = \frac{1}{{x_{1}^{{}} + \sqrt 2 x_{2} }}P - \sigma \le 0 \hfill \\ \;\;\;\;\;\;\;\;0 \le x_{1} ,x_{2} \le 1 \hfill \\ \end{gathered}$$

The comparison of best solution result for this problem is displayed in Table 10. As can be observed, the results given by Tsai^48^ are not feasible while MSCA is significantly better than the results reported in other competitive algorithms. From the comparative results, it is possible to conclude that that MSCA is work well over other methods.

**Table 10:** The best solution for three-bar truss design problem.

Algorithm	*x*_1_	*x*_2_	*g*_1_	*g*_2_	*g*_3_	*f*(***x***)
Hernández^49^	0.788	0.408	NA	NA	NA	263.9
Ray and Saini^50^	0.795	0.395	−0.00169	−0.26124	−0.74045	264.3
Raj et al.^51^	0.78976441	0.40517605	−7.084 × 10^–9^	−1.4675992	−0.53240078	263.89671
Tsai^48^	0.788	0.408	0.00082	−0.2674	−0.73178	263.68^*a*^
Gandomi et al.^37^	0.78867	0.40902	−0.00029	−0.26853	−0.73176	263.9716
MSCA	**0.788690415**	**0.408205144**	**−5.2854 × 10**^**–8**^	**−1.464150692**	**−0.53584936**	**263.89585052**

*a* means not infeasible; NA means not available. Significant values are in bold.

### Case II: Vertical deflection I beam problem

**Figure 10:**
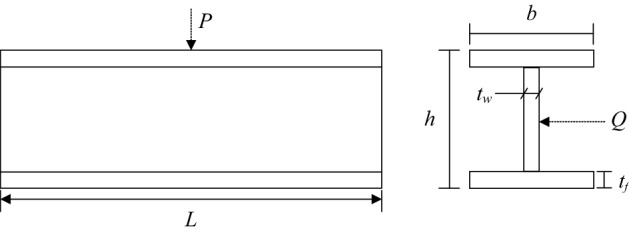
The vertical deflection of an I-beam.

The I-beam structure is one of the design problems for prefabricated buildings with an H-shaped beam in cross-sectional form, as illustrated in Fig. 10. The objective of this problem is to minimize the vertical deflection of an I-beam while satisfying the cross-sectional area and stress constraints for a given load^52^. Consider the variable ***x*** = (*x*_1_, *x*_2_, *x*_3_, *x*_4_) = (*h*, *l*, *t*, *b*), the mathematical formulation of the problem is defined as follows:

7 $$\begin{gathered} {\text{min}}\;\;f({\varvec{x}}) = \frac{5000}{{\frac{{t_{w} \left( {h - 2t_{f} } \right)^{3} }}{12} + \frac{{bt_{f}^{3} }}{6} + 2bt_{f} \left( {\frac{{h - t_{f} }}{2}} \right)^{2} }} \hfill \\ \;\;\;s.t.\;g_{1} \left( {\varvec{x}} \right) = 2bt_{f} + t_{w} \left( {h - 2t_{f} } \right) \le 300 \hfill \\ \;\;\;\;\;\;\;\;g_{2} \left( {\varvec{x}} \right) = \frac{{18h \times 10^{4} }}{{t_{w} \left( {h - 2t_{f} } \right)^{3} + 2bt_{f} \left( {4t_{f}^{2} + 3h\left( {h - 2t_{f} } \right)} \right)}} + \frac{{15b \times 10^{4} }}{{\left( {h - 2t_{f} } \right)t_{w}^{3} + 2t_{f} b^{3} }} \le 6 \hfill \\ \;\;\;\;\;\;\;\;10 \le h \le 80\;\;;\;\;10 \le b \le 50\;\;;\;\;0.9 \le t_{w} \le 5\;\;;\;\;0.9 \le t_{f} \ne 5 \hfill \\ \end{gathered}$$

The best solution and statistical results of this problem are listed in Tables 11 and 12, respectively. From Table 11, it can be seen that MSCA obtain the same objective function value as CSA and get better results than ARSM, Improved ARSM and CS methods. It is possible to conclude in Table 12 that again MSCA has noteworthy performance in all metrics.

**Table 11:** The best solution for vertical deflection of an I-beam problem.

Algorithm	*h*	*b*	*t*_*w*_	*t*_*f*_	*f*(***x***)
ARSM^53^	80	37.05	1.71	2.31	0.0157
Improved ARSM^53^	79.99	48.42	0.9	2.4	0.131
CS^37^	80	50	0.9	2.3216715	0.0130747
CSA^54^	**80**	**49.99999999**	**0.9**	**2.3217923**	**0.01307412**
MSCA	**80**	**50**	**0.900000012**	**2.32179198**	**0.01307412**

Significant values are in bold.

**Table 12:** Statistical result for vertical deflection of an I-beam problem.

Algorithm	Best	Median	Mean	Worst	STD
CS^37^	0.0130747	N/A	0.0132165	0.01353646	1.345 × 10^–4^
CSA^54^	**0.01307412**	0.013091263	0.0131397	0.01339323	9.3686 × 10^–5^
MSCA	**0.01307412**	**0.013074137**	**0.01307415**	**0.01307421**	**2.59 × 10**^**–8**^

Significant values are in bold.

### Case III: Tension string design problem.

**Figure 11:**
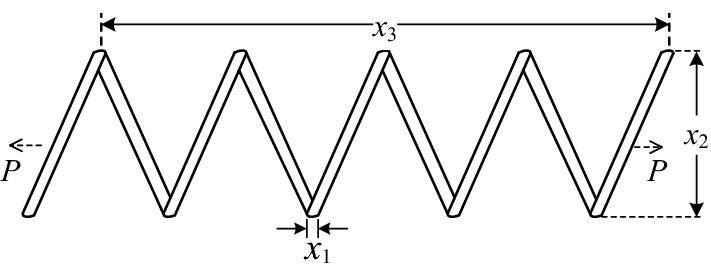
Tension string design.

The main goal of this problem is to minimizes the weight of the tension string with variable include wire diameter(*d*), mean coil diameter(*D*) and the number of active coils(*n*), as shown in Fig. 11. the mathematical model for this problem is given as follows:

8 $$\begin{gathered} {\text{min}}\;\;f({\varvec{x}}) = \left( {x_{3} + 2} \right)x_{2} x_{1}^{2} ,\;\;\;\;\;\;\;\;\;{\varvec{x}} = \left( {x_{1} ,x_{2} ,x_{3} } \right) = \left( {d,D,n} \right) \\ \;\;\;s.t.\;\;g_{1} \left( {\varvec{x}} \right){ = }\;1 - \frac{{x_{2}^{2} x_{3} }}{{71785x_{1}^{4} }} \le 0 \\ \;\;\;\;\;\;\;\;\;g_{2} \left( {\varvec{x}} \right)\;{ = }\frac{{4x_{2}^{2} - x_{1} x_{2} }}{{12566\left( {x_{2} x_{1}^{3} - x_{1}^{4} } \right)}} + \frac{1}{{5108x_{1}^{2} }} - 1 \le 0 \\ \;\;\;\;\;\;\;\;\;g_{3} \left( {\varvec{x}} \right){ = }1 - \frac{{140.45x_{1} }}{{x_{2}^{2} x_{3} }} \le 0 \\ \;\;\;\;\;\;\;\;\;g_{4} \left( {\varvec{x}} \right){ = }\frac{{x_{1} + x_{2} }}{1.5} - 1 \le 0\;\; \\ \;\;0.05 \le x_{1} \le 2;\;\;0.25 \le x_{2} \le 1.3;\;\;2 \le x_{3} \le 15 \\ \end{gathered}$$

The comparison results with respect to the best solution between MSCA and existing algorithms are presented in Table 13, and the related statistical information is listed in Table 14. Based on both tables, MSCA exhibits more competitive results than other methods, indicating that the proposed MSCA algorithm has strong engineering practical value.

**Table 13:** The best solution for tension string design problem.

Algorithm	*x*_1_	*x*_2_	*x*_3_	*f*(***x***)
Belegundu^55^	0.05	0.3159	14.25	0.0128334
Coello^56^	0.05148	0.351661	11.632201	0.01270478
Ray and Saini^50^	0.050417	0.321532	13.979915	0.01306
Coello and Montes^57^	0.051989	0.363965	10.890522	0.012681
Ray and Liew ^58^	0.052160217	0.368158695	10.64844226	0.012669249
Raj et al.^51^	0.053862	0.41128365	8.6843798	0.0127484
Mahdavi et al.^59^	0.05115438	0.34987116	12.0764321	0.0126706
He and Wang^60^	0.051728	0.357644	11.244543	0.0126747
Montes and Coello^61^	0.051643	0.35536	11.397926	0.012698
Kaveh and Talatahari^62^	0.051865	0.3615	11	0.0126432^*a*^
Coelho^63^	0.051515	0.352529	11.538862	0.012665^*a*^
Akay and Karaboga^64^	0.051749	0.358179	11.203763	0.012665^*a*^
MSCA	**0.051781993**	**0.358944836**	**11.16078852**	**0.012666807**

*a* means infeasible. Significant values are in bold.

**Table 14:** Statistical result for tension string design problem.

Algorithm	Best	Median	Mean	Worst	STD
Belegundu^55^	0.0128334	NA	NA	NA	NA
Coello^56^	0.01270478	0.01275576	0.0127692	**0.01282208**	**3.9390 × 10**^**–5**^
Ray and Saini^50^	0.01306	NA	0.015526	0.018992	NA
Coello and Montes^57^	0.012681	NA	0.012742	0.012973	5.9000 × 10^–5^
Ray and Liew^58^	0.01266925	0.01292267	0.01292267	0.01671727	5.92 × 10^–4^
He and Wang^60^	0.0126747	NA	**0.01273**	0.012924	5.1985 × 10^–5^
Montes and Coello^61^	0.012698	NA	0.013461	0.16485	9.6600 × 10^–4^
Kaveh and Talatahari^62^	0.0126432^a^	NA	0.01272^a^	0.012884^a^	3.4888 × 10^–5^
Coelho^63^	0.012665^a^	0.012957^a^	0.013524^a^	0.017759^a^	0.001268
Akay and Karaboga^64^	0.012665^a^	NA	0.012709^a^	NA	0.012813
MSCA	**0.01266681**	**0.01273110**	0.01281752	0.01334238	1.90 × 10^–4^

NA means not available; *a* means infeasible. Significant values are in bold.

### Case IV: Welded beam design problem

**Figure 12:**
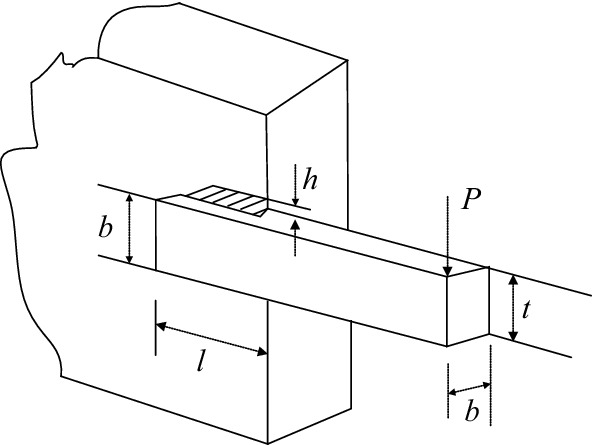
Welded beam design.

Welded beam design is an important part of the engineering design problem, as shown in Fig. 12, the objective is to minimize the costs of welded beam^51^ by selecting four variables: the thickness of weld *h*, length of welded joint *l*, width of the beam *t* and thickness of the beam *b*. The optimization model with variable ${\varvec{x}} = \left( {x_{1} ,x_{2} ,x_{3} ,x_{4} } \right) = \left( {h,l,t,b} \right)$ is expressed as follows:

#### Version I:

9 $$\begin{gathered} {\text{min}}\;\;f({\varvec{x}}) = 1.10471x_{1}^{2} x_{2} + 0.04811x_{3} x_{4} (14 + x_{2} ) \\ \;\;\;s.t.\;\;g_{1} \left( {\varvec{x}} \right)\;{ = }\;\tau \left( {\varvec{x}} \right) - \tau_{\max } \le 0 \\ \;\;\;\;\;\;\;\;\;g_{2} \left( {\varvec{x}} \right)\;{ = }\;\sigma \left( {\varvec{x}} \right) - \sigma_{\max } \le 0 \\ \;\;\;\;\;\;\;\;\;g_{3} \left( {\varvec{x}} \right)\;{ = }\;x_{1} - x_{4} \le 0;\;\;\;\;\;\;\;\; \\ \;\;\;\;\;\;\;\;\;g_{4} \left( {\varvec{x}} \right)\;{ = }\;{0}{\text{.125 - }}x_{1} \le 0 \\ \;\;\;\;\;\;\;\;\;g_{5} \left( {\varvec{x}} \right)\;{ = }\;\delta \left( {\varvec{x}} \right) - 0.25 \le 0 \\ \;\;\;\;\;\;\;\;\;g_{6} \left( {\varvec{x}} \right)\;{ = }\;P - P_{c} \le 0 \\ \;0.1 \le x_{1} ,x_{4} \le 2\;\;;\;\;0.1 \le x_{2} ,x_{3} \le 10 \\ \end{gathered}$$

where the shear stress $\tau$ is defined as follows:

10 $$\tau \;{ = }\;\sqrt {\tau_{1}^{2} + 2\tau_{1} \tau_{2} \left( {\frac{{x_{2} }}{2R}} \right) + \tau_{2}^{2} } ;\;\tau_{1} = \frac{P}{{x_{1} x_{2} \sqrt 2 }};\;\tau_{2} = \frac{MR}{J}$$

11 $$M\;{ = }\;P\left( {L + \frac{{x_{2} }}{2}} \right);\;J = 2\left\{ {\frac{{x_{1} x_{2} }}{\sqrt 2 }\left[ {\frac{{x_{2}^{2} }}{12} + \left( {\frac{{x_{1} + x_{3} }}{2}} \right)^{2} } \right]} \right\}$$

12 $$R\;{ = }\;\sqrt {\frac{{x_{2}^{2} }}{4} + \left( {\frac{{x_{1} + x_{3} }}{2}} \right)^{2} } ;\;\sigma = \frac{6PL}{{x_{1} x_{3}^{2} }};\;\delta \;{ = }\;\frac{{4PL^{3} }}{{Ex_{3}^{3} x_{4} }}$$

13 $$P_{c} = \frac{{4.013\sqrt {\frac{{EGx_{3}^{2} x_{4}^{6} }}{36}} }}{{L^{2} }}\left( {1 - \frac{{x_{3} }}{2L}\sqrt{\frac{E}{4G}} } \right)$$

14 $$G = 12 \times 10^{6} {\text{psi}},\;E = 30 \times 10^{6} {\text{psi}},\;P = 6000{\text{lb}},\;L = 14{\text{in}}$$

The MSCA is compared with four optimization methods, and the best solution and statistical results are reported in Tables 15 and 16. the results indicate that MSCA obtained the solution more accurately and more competitive than other methods.

**Table 15:** The best solution for welded beam design problem on version I.

Algorithm	*x*_1_	*x*_2_	*x*_3_	*x*_4_	*f*(***x***)
Ragsdell and Phillips^65^	0.2455	6.196	8.273	0.2455	2.385937
Rao^66^	0.2455	6.196	8.273	0.2455	2.386
Ray and Liew^58^	0.244438276	6.237967234	8.288576143	0.244566182	2.3854347
Hwang and He^67^	0.2231	1.5815	12.8468	0.2245	2.25^*a*^
MSCA	**0.244249519**	**6.206365305**	**8.312174308**	**0.24432385**	**2.383286722**

*a* means infeasible. Significant values are in bold.

**Table 16:** Statistical result for welded beam design problem on version I.

Algorithm	Best	Median	Mean	Worst	STD
Ragsdell and Phillips^65^	2.385937	NA	NA	NA	NA
Rao^66^	2.386	NA	NA	NA	NA
Ray and Liew^58^	2.3854347	3.0025883	3.2551371	6.3996785	0.959078
Hwang and He^67^	2.25^a^	NA	2.26^a^	2.28^a^	NA
MSCA	**2.383286722**	**2.387845945**	**2.388104097**	**2.393825358**	**0.002758472**

NA means not available; *a* means infeasible. Significant values are in bold.

#### Version II

In version II, the researchers modified the definitions of some items on the basis of version I and added a new constraint $g_{7}$, which are defined as follows:

15 $$g_{7} \left( {\varvec{x}} \right)\;{ = }\;0.10471x_{1}^{2} + 0.04811x_{3} x_{4} (14 + x_{2} ) - 5 \le 0$$

16 $$J = 2\left\{ {\sqrt 2 x_{1} x_{2} \left[ {\frac{{x_{2}^{2} }}{4} + \left( {\frac{{x_{1} + x_{3} }}{2}} \right)^{2} } \right]} \right\};\;\sigma = \frac{{6PL^{3} }}{{x_{1} x_{3}^{3} x_{4} }}$$

17 $$P_{c} = \frac{{4.013E\sqrt {\frac{{x_{3}^{2} x_{4}^{6} }}{36}} }}{{L^{2} }}\left( {1 - \frac{{x_{3} }}{2L}\sqrt{\frac{E}{4G}} } \right)$$

Tables 17 and 18 summarize the best solution and statistical results for version II, respectively. It can be seen from the simulation results that MSCA can still obtain convincing results with the addition of constraint of $g_{7}$, indicating that the proposed MSCA performs better than existing studies.

**Table 17:** The best solution for welded beam design problem on version II.

Algorithm	*x*_1_	*x*_2_	*x*_3_	*x*_4_	*f*(***x***)
Coello^56^	0.2088	3.4205	8.9975	0.21	1.748309
Coello and Montes^57^	0.205986	3.471328	9.020224	0.20648	1.728226
He and Wang^60^	0.202369	3.544214	9.04821	0.205723	1.728024
Dimopoulos^68^	0.2015	3.562	9.041398	0.205706	1.731186
Mahdavi et al.^59^	0.20573	3.47049	9.03662	0.20573	1.7248
Montes et al.^69^	0.20573	3.470489	9.036624	0.20573	1.724852
Montes and Coello^61^	0.199742	3.61206	9.0375	0.206082	1.7373
Cagnina et al.^70^	0.205729	3.470488	9.036624	0.205729	1.724852
Kaveh and Talatahari^71^	0.205729	3.469875	9.036805	0.205765	1.724849
Kaveh and Talatahari^62^	0.2057	3.471131	9.036683	0.205731	1.724918
Gandomi et al.^72^	0.2015	3.562	9.0414	0.2057	1.73121
Mehta and Dasgupta^73^	0.20572885	3.47050567	9.03662392	0.20572964	1.724855
Akay and Karaboga^64^	0.20573	3.470489	9.036624	0.20573	1.724852
MSCA	**0.205187143**	**3.266067065**	**9.03380051**	**0.205913062**	**1.69710013**

Significant values are in bold.

**Table 18:** Statistical result for welded beam design problem on version II.

Algorithm	Best	Median	Mean	Worst	STD
Coello^56^	1.748309	NA	1.771973	1.785835	0.01122
Coello and Montes^57^	1.728226	NA	1.792654	1.993408	0.07471
He and Wang^60^	1.728024	NA	1.748831	1.782143	0.012926
Dimopoulos^68^	1.731186	NA	NA	NA	NA
Montes et al.^69^	1.724852	NA	1.725	NA	1.0 × 10^–15^
Montes and Coello^61^	1.7373	NA	1.81329	1.994651	0.0705
Cagnina et al.^70^	1.724852	NA	2.0574	NA	0.2154
Kaveh and Talatahari^71^	1.724849	NA	1.727564	1.759522	0.008254
Kaveh and Talatahari^62^	1.724918	NA	1.729752	1.775961	0.0092
Gandomi et al.^72^	1.7312065	NA	1.878656	2.3455793	0.2677989
Mehta and Dasgupta^73^	1.724855	1.724861	1.724865	1.72489	NA
Akay and Karaboga^64^	1.724852	NA	1.741913	NA	0.031
MSCA	**1.69710013**	**1.700828362**	**1.70209677**	**1.722162002**	**0.005815737**

NA means not available. Significant values are in bold.

### Case V: Gear train design problem

**Figure 13:**
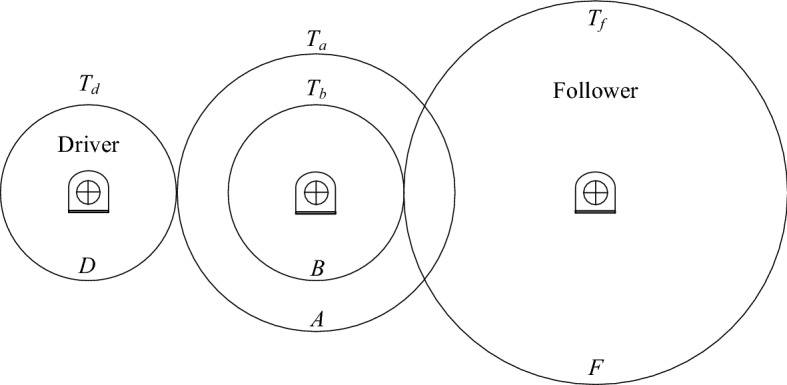
Gear train design.

The gear train design problem was first proposed by Sandgren^74^ as an unconstrained optimization problem, presented in Fig. 13. The task of the problem is to find the best variable vector ${\varvec{x}} = \left( {x_{1} ,x_{2} ,x_{3} ,x_{4} } \right) = \left( {T_{d} ,T_{b} ,T_{a} ,T_{f} } \right)$ to minimize cost of gear ratio. The optimization formula is given as follows:

18 $$\begin{gathered} {\text{min}}\;\;f({\varvec{x}}) = \left( {\frac{1}{6.931} - \frac{{x_{1} x_{2} }}{{x_{3} x_{4} }}} \right)^{2} \hfill \\ \;\;\;s.t.\;\;12 \le x_{i} \le 60,i = 1,2,3,4 \hfill \\ \;\;\;\;\;\;\;\;\;x_{i} \in Z^{ + } ,i = 1,2,3,4 \hfill \\ \end{gathered}$$

where gear ratio = $\frac{{x_{1} x_{2} }}{{x_{3} x_{4} }}$.

To address this issue, MSCA was compared with several classical methods, and the best solutions recorded for MSCA and other literature results are listed in Table 19. On the other hand, the corresponding statistical results are represented in Table 20. The both tables show that MSCA provides more stable and accurate solutions than other methods.

**Table 19:** The best solution for gear train design problem.

Algorithm	*T*_*d*_(*x*_1_)	*T*_*b*_(*x*_2_)	*T*_*a*_(*x*_3_)	*T*_*f*_(*x*_4_)	Gear ratio	*f*(***x***)
Sandgren^75^	18	22	45	60	0.146667	5.712 × 10^–6^
Kannan and Kramer^76^	13	15	33	41	0.144124	2.146 × 10^–8^
Deb and Goyal^77^	19	16	49	43	0.144281	2.701 × 10^–12^
Gandomi et al.^37^	19	16	43	49	0.144281	2.701 × 10^–12^
Garg^78^	**19**	**16**	**43**	**49**	**0.14428096**	**2.7008571 × 10**^**–12**^
MSCA	**19**	**16**	**43**	**49**	**0.14428096**	**2.7008571 × 10**^**–12**^

Significant values are in bold.

**Table 20:** Statistical result for gear train design problem.

Algorithm	Best	Median	Mean	Worst	STD
Gandomi et al.^78^	2.7009 × 10^–12^	NA	1.9841 × 10^–9^	2.3576 × 10^–9^	3.5546 × 10^–9^
Garg^37^	**2.7008571 × 10**^**–12**^	9.9215795 × 10^–10^	1.2149276 × 10^–9^	3.2999231 × 10^–9^	8.77 × 10^–10^
MSCA	**2.7008571 × 10**^**–12**^	**2.3078157 × 10**^**–11**^	**4.8092363 × 10**^**–11**^	**3.0675559 × 10**^**–10**^	**7.4270038 × 10**^**–11**^

NA means not available. Significant values are in bold.

### Case VI: Pressure vessel design problem

**Figure 14:**
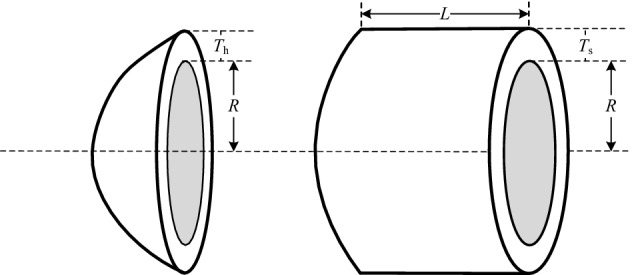
Pressure vessel design.

IN this problem, shown in Fig. 14, the task is to minimize the total cost of the vessel with decision vector ${\varvec{x}}$ = $\left( {x_{1} ,x_{2} ,x_{3} ,x_{4} } \right)$ = $\left( {T_{s} ,T_{h} ,R,L} \right)$^79^. The optimization model is constructed as follows:

19 $$\begin{gathered} {\text{min}}\;\;f({\varvec{x}}) = 0.6224x_{1} x_{3} x_{4} + 1.7781x_{2} x_{3}^{2} + 3.1661x_{1}^{2} x_{4} + 19.84x_{1}^{2} x_{3} \hfill \\ \;\;\;s.t.\;\;g_{1} ({\varvec{x}}) = - x_{1} + 0.0193x_{3} \le 0 \hfill \\ \;\;\;\;\;\;\;\;\;g_{2} ({\varvec{x}}) = - x_{2} + 0.00954x_{3} \le 0 \hfill \\ \;\;\;\;\;\;\;\;\;g_{3} ({\varvec{x}}) = - \pi x_{3}^{2} x_{4} + \frac{4}{3}\pi x_{3}^{3} + 1,296,000 \le 0 \hfill \\ \;\;\;\;\;\;\;\;\;g_{4} ({\varvec{x}}) = x_{4} - 240 \le 0 \hfill \\ \end{gathered}$$

#### Version I:

20 $$0.0625 \le x_{1} ,x_{2} \le 99 \times 0.0625\;;\;10 \le x_{3} ,x_{4} \le 200$$

The best results and statistical results for version I are reported in Tables 21 and 22, respectively. As can be seen from Table 21, MCSA obtain more objective results than other literature, and from Table 22, MCSA further obtains a more concentrated solution distribution, demonstrating the superior search performance of the proposed method.

**Table 21:** The best solution for pressure vessel design problem on version I.

Algorithm	*x*_1_	*x*_2_	*x*_3_	*x*_4_	*f*(***x***)
Sandgren^74^	1.125	0.625	47.7	117.701	8129.1036
Kannan and Kramer^80^	1.125	0.625	58.291	43.69	7198.0428
Coello and Montes^57^	0.8125	0.4375	42.097398	176.65405	6059.946
He and Wang^60^	0.8125	0.4375	42.091266	176.7465	6061.0777
Montes and Coello^61^	0.8125	0.4375	42.098087	176.640518	6059.7456
Coelho^63^	0.8125	0.4375	42.0984	176.6372	6059.7208
He et al.^81^	0.8125	0.4375	42.098445	176.636595	6059.7143
Montes et al.^69^	0.8125	0.4375	42.098446	176.636047	6059.70166
Gandomi et al.^37^	0.8125	0.4375	42.0984456	176.6365958	6059.714335
Akay and Karaboga^64^	0.8125	0.4375	42.098446	176.636596	6059.714339
MSCA	**0.780583407**	**0.3917558**	**40.4190779**	**198.964126**	**5917.509793**

Significant values are in bold.

**Table 22:** Statistical result for pressure vessel design problem on version I.

Algorithm	Best	Median	Mean	Worst	STD
Sandgren^74^	8129.1036	NA	N/A	N/A	N/A
Kannan and Kramer^80^	7198.0428	NA	N/A	N/A	N/A
Coello and Montes^57^	6059.9463	NA	6177.2533	6469.322	130.9297
Montes and Coello^61^	6059.7456	NA	6850.0049	7332.8798	426
Gandomi et al.^37^	6059.714	NA	6447.736	6495.347	502.693
Coelho^63^	6059.7208	6257.5943	6440.3786	7544.4925	448.4711
He et al.^81^	6059.7143	NA	6289.92881	NA	305.78
Akay and Karaboga^64^	6059.714339	NA	6245.30814	NA	205
MSCA	**5917.509793**	**5994.4224**	**6029.24374**	**6396.551211**	**113.2470944**

NA means not available. Significant values are in bold.

#### Version II:

21 $$0.0625 \le x_{1} ,x_{2} \le 99 \times 0.0625\;;\;10 \le x_{3} \le 200\;;\;10 \le x_{4} \le 240$$

In version II, the upper bound of the decision variable *x*_4_ is set as 240, the best results and statistical results summarized by MSCA and control methods for version II in Tables 23 and 24, respectively. Analysis from both tables, MSCA reveals better performance than the existing studies. The comparison results further prove that MCSA has better feasibility in engineering than the other approaches.

**Table 23:** The best solution for pressure vessel design problem on version II.

Algorithm	*x*_1_	*x*_2_	*x*_3_	*x*_4_	*f*(***x***)
Dimopoulos^68^	0.75	0.375	38.8601	221.36549	5850.38306
Mahdavi et al.^59^	0.75	0.375	38.8601	221.36553	5849.76169
Gandomi et al.^72^	0.75	0.375	38.8601	221.36547	5850.38306
MSCA	**0.73822151**	**0.36818507**	**38.16830296**	**232.73616127**	**5849.52062**

Significant values are in bold.

**Table 24:** Statistical result for pressure vessel design problem on version II.

Algorithm	Best	Median	Mean	Worst	STD
Dimopoulos^68^	5850.383060	NA	NA	NA	NA
Mahdavi et al.^59^	5849.761700	NA	NA	NA	NA
Gandomi et al.^72^	5850.383060	NA	**5937.337900**	6258.968250	164.547470
MSCA	**5849.520622**	**5919.655417**	5944.332084	**6217.813607**	**87.356362**

NA means not available. Significant values are in bold.

## Conclusion

In this article, the position update adjustment strategy and Levy random walk mutation mechanism were adopted into the original SCA to create the proposed MCSA. The position update adjustment strategy considers the swarm search for potentially better solutions around the current individual and the global optimal individual, effectively expanding the search and improving the convergence speed of the original SCA. And the Levy random walk mutation mechanism effectively increases the original SCA diversity to ensure that the SCAs jump out of the local optimal position and improve the search accuracy.

For qualitative analysis of proposed method (MSCA), it has been passed two levels of benchmark test suites—the classic and the IEEE CEC2017 function. box plots and convergence curves were employed to verify the performance of robustness and convergence. In addition, Wilcoxon signed-rank test and ranksum test were adopted to verify statistical significance. The experimental results show that MSCA is able to balance the exploration and exploitation process. Finally, MSCA is used to solve six complex real-world engineering design problems, and the comparison with the results of several other state-of-the-art methods proves that the proposed method can achieve competitive results. Based on the convincing results obtained from existing research, in the future we will try to apply the proposed method to image recognition and time series forecasting problems.

## Data Availability

The data that support the findings of this study are available from the corresponding author upon request. There are no restrictions on data availability.
